# Dendritic Cell Dysfunction Underlies Immune Escape After Adoptive Cellular Therapy in Glioblastoma

**DOI:** 10.3390/cancers18162669

**Published:** 2026-08-18

**Authors:** Dan Jin, Bayli DiVita, Alexandra Reid, Caitland Love, John W. Figg, Connor Francis, Laura Falceto Font, Kaytora Long-James, David Hilferty, Sofia Stansbury, Norman Morikawa, Mathew Sebastian, Steeve Boulant, Duane A. Mitchell, Catherine Flores

**Affiliations:** 1 University of Florida Brain Tumor Immunotherapy Program, Preston A. Wells, Jr. Center for Brain Tumor Therapy, Lillian S. Wells Department of Neurosurgery, McKnight Brain Institute, University of Florida, Gainesville, FL 32610, USA; dan.jin@ufl.edu (D.J.);; 2 Department of Molecular Genetics & Microbiology, College of Medicine, University of Florida, Gainesville, FL 32611, USA

**Keywords:** dendritic cell dysfunction, adoptive cellular therapy, hypoxia pathway, immune evasion

## Abstract

**Background/Objectives**: Glioblastoma (GBM) remains a lethal primary CNS malignancy with limited response to immunotherapy. Adoptive cellular therapy (ACT) improves survival in preclinical models, yet tumors ultimately recur. While T cell exhaustion is a common mechanism of resistance, the contribution of dendritic cell (DC) dysfunction remains unclear. We aimed to define mechanisms of immune escape following ACT, focusing on DC function and the role of hypoxia. **Methods**: Using a murine glioma model (KR158B-luc), mice were treated with ACT consisting of tumor RNA-pulsed DC vaccines and adoptively transferred T cells. Tumor-infiltrating immune populations were analyzed by flow cytometry. DC function was assessed using T cell activation assays. Bulk RNA sequencing and gene set enrichment analysis were performed on sorted DCs. Hypoxia was modeled in vitro, and HIF1α was perturbed using CRISPR-mediated knock-out. **Results**: ACT significantly increased survival but did not prevent tumor recurrence. Escaped tumors contained abundant cytotoxic, non-exhausted T cells, indicating that T cell dysfunction was not the primary driver of recurrence under ACT. Instead, tumor-associated DCs exhibited impaired T cell activation despite preserved antigen uptake. Transcriptomic analyses revealed reduced antigen presentation and co-stimulatory signaling, alongside increased expression of tolerogenic factors. ACT-treated tumors demonstrated heightened hypoxia pathway activation, with elevated HIF1α expression in DCs. Hypoxia induced DC tolerogenic programs and reduced their ability to activate T cells, an effect partially reversed by HIF1α disruption. Increased immune infiltration and inflammation following ACT further amplified hypoxia signaling and enhanced DC tolerance. **Conclusions**: DC dysfunction is one of the key mechanisms of immune escape following ACT in glioma. Hypoxia-driven tolerization of DCs impairs sustained anti-tumor immunity, highlighting the hypoxia–DC axis as a promising therapeutic target to enhance immunotherapy efficacy.

## Simple Summary

Adoptive cellular therapy (ACT) improves survival in preclinical models, yet tumors ultimately recur. The question remains why ACT failed to give a long-lasting survival benefit. Our study uncovers a mechanism of DC dysfunction involved in the failure to sustain a long-term anti-tumor immune response. The study highlights the role of ACT treatment-triggered stimuli in inducing DC tolerance. The treatment triggered stimuli include elevated hypoxia pathway activation and secreted factors from immune–tumor reactions. These findings provide new insights into immune evasion from immunotherapy.

## 1. Introduction

Glioblastoma (GBM) is the most common primary central nervous system (CNS) malignancy in adults and remains associated with a median overall survival of less than 15 months [1]. Despite the transformative success of immunotherapies across multiple cancer types, clinical benefit in GBM has been limited. This lack of efficacy is attributed to several factors, including the immune-specialized nature of the CNS, extensive intra- and inter-tumoral heterogeneity, and a profoundly immunosuppressive tumor microenvironment [2,3]. Elucidating the mechanisms underlying immunotherapy resistance is therefore essential for the development of more effective therapeutic strategies for GBM.

Dendritic cells (DCs) are central to the initiation and propagation of anti-tumor immunity through antigen uptake, processing, and presentation to T cells [4]. In the context of cancer, DCs play a critical role in addressing tumor antigen heterogeneity by priming diverse T cell repertoires and facilitating antigen spreading within secondary lymphoid organs, thereby promoting sustained anti-tumor responses [5]. However, tumors can disrupt DC biology at multiple levels, impairing their differentiation, function, and survival [6,7]. Tumor-associated DCs often acquire a tolerogenic phenotype characterized by the expression of immunosuppressive mediators, including IL-10, TGF-β, indoleamine 2,3-dioxygenase (IDO), inducible nitric oxide synthase (iNOS), and arginase. This phenotypic shift results in suboptimal T cell priming and the expansion of regulatory T cells, ultimately reinforcing immunosuppression [8]. These mechanisms represent significant barriers to effective immunotherapy.

Hypoxia is a defining feature of solid tumors, arising from the imbalance between rapid cellular proliferation and insufficient vascular supply [9]. Hypoxic signaling pathways exert broad effects on immune cell function under both physiological and pathological conditions [10]. While physiological hypoxia contributes to immune homeostasis, pathological hypoxia within the tumor microenvironment can dysregulate immune responses and promote tumor progression [10]. The impact of hypoxia on DC biology, however, remains incompletely defined. Some studies report that hypoxia impairs DC maturation, antigen presentation, and T cell priming [11,12,13], whereas others suggest it may enhance DC migration and activation [14,15]. These conflicting observations underscore the context-dependent nature of hypoxia-mediated immune regulation and highlight the need for further mechanistic investigation.

Adoptive cellular therapy (ACT) has demonstrated significant survival benefit in preclinical glioma models; however, durable responses are limited, with most tumors ultimately recurring. Prior work from our group identified immune editing and tumor antigen shift as key mechanisms of escape following ACT [16]. In the present study, we identify dendritic cell dysfunction as an additional key player in the failure of maintaining a long-term anti-tumor immune response. We show that ACT induces immune infiltration and inflammatory reactions within the tumor microenvironment, which is accompanied by increased activation of hypoxia-related pathways. This enhanced hypoxia pathway activation and secreted factors from immune–tumor reactions promote the acquisition of a stronger tolerogenic phenotype in ACT-escaped tumor-associated DCs, resulting in impaired antigen presentation and reduced capacity to prime novel T cell responses against evolving shifted tumor antigens. Notably, adoptively transferred T cells retain cytotoxic function and do not exhibit features of exhaustion, suggesting that failure of sustained tumor control is driven, at least in part, by defective DC-mediated propagation of the anti-tumor immune response.

## 2. Methods

### 2.1. Cell Lines and Animals

KR158B gliomas cell line is derived from a GEMM model expressing germline global NF1−/+ and TP53−/+ modifications. Tumor development in vivo results in the loss of NF1 and TP53 wildtype alleles, leading to homozygous NF1 and TP53 deletion [17,18]. KR158B gliomas cell line with TP53 and NF1 knock-out was supplied by Dr. Karlyne M. Reilly at the National Cancer Institute, Bethesda, MD, USA [17]. KR158B was transduced with luciferase gene containing lentivirus to generate KR158B-luc. KR158B-luc-OVA was generated by infecting KR158B-luc with ovalbumin (OVA) gene expressing lentivirus. Female 5–8-week-old C57BL/6 mice (The Jackson Laboratory, Gainesville, FL, USA, stock 000664), OT1 (The Jackson Laboratory, Gainesville, FL, USA, stock 003831), CD45.1 (Jackson Laboratories stock 002014), C57BL/6-Tg (UBC-GFP) 30Scha/J (The Jackson Laboratory, Gainesville, FL, USA, stock 004353) were used for experiments. The investigators adhered to the “Guide for the Care and Use of Laboratory Animals” as proposed by the committee on care of Laboratory Animal Resources Commission on Life Sciences, National Research Council. The facilities at the University of Florida Animal Care Services are fully accredited by the American Association for Accreditation of Laboratory Animal Care, and all studies were approved by the University of Florida Institutional Animal Care and Use Committee.

### 2.2. Bone Marrow-Derived Dendritic Cell (BMDC) Generation and Electroporation

Bone marrow was harvested from C57BL/6 mice, red blood cells were lysed and MNCs (mononuclear cells) were cultured in dendritic differentiation medium containing GM-CSF (10 ng/mL, R&D Systems, Minneapolis, MN, USA, cat# 415-ML/CF) and IL-4 (10 ng/mL, R&D Systems, Minneapolis, MN, USA, cat# 404ML/CF) for 3 days. Medium is replaced with fresh dendritic cell differentiation medium on day 3, and floating or loosely attached dendritic cells were harvested on day 7 for experimental use. Electroporation was done by BTX Single Waveform Electroporation System (Harvard Apparatus, Holliston, MA, USA, ECM 830) at settings at LV mode, desired field strength 1500 V/cm, 500 μs, 1 pulse. For total tumor RNA electroporation, 25 μg total RNA is mixed with 5 million BMDCs in 200 ul Opti-MEM in 2 mm electroporation Cuvette (Harvard Apparatus BTX, Holliston, MA, USA, cat# 58017-895). Culture electroporated BMDCs for additional 24 h before vaccine or co-culture with T cells.

### 2.3. Normoxia and Hypoxia Treatment

BMDCs were cultured for indicated time at normoxia (atmospheric 37 °C, 21% O_2_, 5% CO_2_) or hypoxia (37 °C, 1% O_2_, 5% CO_2_) in the Xvivo hypoxia hood and culture combo workstation (BioSpherix X3, Parish, NY, USA).

### 2.4. Tumor-Reactive T Cell Generation

Total RNA was isolated from KR158B-luc tumor and electroporated into BMDCs using BTX Single Waveform Electroporation System. Naïve mice received intradermal vaccination with 250,000 total tumor RNA-pulsed DCs. Spleens were harvested 7 days later, and the splenocytes were expanded ex vivo by co-culture with KR158B-luc RNA-pulsed DCs at ratio of 1:10 (DC:splenocytes) in 50 IU/mL mIL-2 (R&D Systems, Minneapolis, MN, USA, cat# 402-ML-020/CF) containing T cell medium [RPMI-1640 (Gibco, Waltham, MA, USA, cat# 11875119), 10% FBS, 55 µM 2-Mercaptoethanol (Gibco, Waltham, MA, USA, cat# 21-985-023), 1 mM Sodium pyruvate (Gibco, 11360-070), 1X Nonessential amino acids (Gibco, 11140-050), 2 mM L-glutamine, (Gibco, 25030164), 100 U/mL Penicillin/Streptomycin (Gibco, 15140148)] for 6 days. Passage cells in 1:2 every 2–3 days.

### 2.5. Tumor Implantation and ACT Treatment

10^4^ KR158B cells prepared in 50% methylcellulose PBS suspension (Fisher Scientific, Gainesville, FL, USA, cat# HSC001) were implanted intracranially by injecting 2 mm lateral to the midline at bregma suture and 3 mm deep in the cortex. Tumors were injected with a stereotactic frame (Stoelting, Wood Dale, IL, USA, cat# 53311) and a 250 μL syringe (Hamilton, Reno, NV, USA, cat# 81120) with a 25-gauge needle for KR158B tumors.

Treatment of tumor-bearing mice began with 9 Gy myeloablation on day 5 post intracranial injection with X-ray irradiation (Precision X-Ray, Madison, CT, USA, X-RAD 320). On day 6 post intracranial tumor injection, mice received a single intravenous injection with 10^7^ autologous ex vivo expanded TTRNA T cells with either 25 × 10^4^ lineage-depleted hematopoietic stem and progenitor cells (Miltenyi Biotec, Gaithersburg, MD, USA, cat# 130-090-858). 2.5 × 10^5^ of tumor RNA-pulsed dendritic cell vaccine were given intradermally. On day 13 and 20, 2.5 × 10^5^ tumor RNA-pulsed dendritic cell vaccines were given respectively as 2nd and 3rd dose.

### 2.6. RNA Sequencing and Analysis

Primary tumors were harvested from endpoint approaching KR158B-luc bearing mice without treatment. ACT-escaped tumors were harvested from endpoint approaching KR158B-luc-bearing mice after ACT treatment. Resting spleens were harvested from naïve mice. Activated spleens were harvested from mice 12 h after intravenously injecting LPS (0.1 µg/g) and OVA (2.5 µg/g). Tumor-associated DCs and splenic DCs were FACS-sorted based on CD45^+^MHCII^+^CD11c^+^ markers. 50,000 cells were sorted and RNA were extracted by RNeasy Plus Micro kit (Qiagen, Germantown MD, USA, cat# 74034). Genomic DNA contamination was removed using Optimal DNA depletion columns (Qiagen). RNA samples were submitted to MedGenome Inc. (Foster City, CA, USA) for ultra-low RNA input sequencing. Three repeats represent 3 biological repeats.

For library preparation, Takara SMART-Seq v4 Ultra-low input RNA kit (Takara Bio, San Jose, CA, USA) was used. Library QC was performed on Tapestation. Bulk RNA-seq was performed using a Novaseq 6000 (Illumina, San Diego, CA, USA) on a 2 × 150 bp sequencing run.

Data quality checking was performed using FastQC (v0.11.9, RRID:SCR_014583). The adapter trimming was performed using fastq-mcf program (v1.05) and cutadapt (v4.7). Removal of unwanted sequences including mitochondrial genome sequences, rRNAs, tRNAs, adapter sequences and others were performed by Bowtie2 (v2.5.3). The paired-end reads were aligned to the reference Mouse genome (mm10) using STAR (2.7.11b). The raw read counts were estimated using HTSeq (v2.0.5). Only reads mapping to a single gene are counted. Read count data were normalized using DESeq2 (1.40.2).

Pathway analysis using GSEA software v4.3.228 from the Broad Institute was used to interrogate biological pathways from the Molecular Signatures Database (m2.all.v2023.1) with classic settings, and normalized counts gene lists were input for analysis. Correlation plots were generated in R 4.3.3 (29 February 2024 ucrt) using ggplot2 (4.0.0) and corrplot package (0.95).

### 2.7. Nanostring GeoMx

Spatial transcriptomics were performed using the nanoString GeoMx^®^ Digital Spatial Profiler (nanoString technologies, Seattle, WA, USA). KR158B-luc tumor brains with 9 Gy + HSC, ACT and HSC + αPD1 treatments were fixed and sectioned at 5 μm thickness. Sliced tissues were shipped to NanoString Technologies for slice staining and processing for GeoMx DSP. After the slides were scanned, 24 geometric regions of interest (ROIs) were selected in CD45^+^ regions from each sample for NGS readout. Detailed procedures of slide staining, the NGS readout could be found on the Nanostring university website. HSC + αPD1 treatment has been described in a previous publication [19] which includes 5Gy total body irradiation followed by 250,000 HSCs transfer and 5 doses of anti-PD1.

### 2.8. RT-PCR

Total RNAs were extracted by RNeasy Mini Kit (Qiagen, Germantown, MD, USA, cat# 74104) and reverse transcribed by iScript™ Reverse Transcription Supermix (BioRad, Hercules, CA, USA, cat# 1708840). qPCR was done by PowerTrack™ SYBR Green Master Mix (Thermal Fisher Scientific, Waltham, MA, USA, cat# A46012). Beta actin gene was used as internal reference gene for normalization. Relative gene expressions are calculated by 2^−ΔΔCt^ method. Primers used in the manuscript are listed in Table S4.

### 2.9. BMDCs CRISPR Knocking Out

HIF1α sgRNA were purchased from Thermo Fisher Scientific (Waltham, MA, USA, cat# A35533). 1.2 μg sgRNA and 3.8 µg Cas9 mRNA (Trilink Biotechnologies, San Diego, CA, USA, cat# L-7606-1000) were mixed with 1.25 million BMDCs in 50 µL OptiMEM in 1 mm electroporation cuvette (BTX, Harvard Apparatus BTX, Holliston, MA, USA, cat# 58017-890). Electroporation was done by BTX Single Waveform Electroporation System (Harvard Apparatus, Holliston, MA, USA, ECM 830) at settings at LV mode, desired field strength 1500 V/cm, 500 μs, 1 pulse. After electroporation, cells were cultured for 72 h before DNA and RNA extraction. Both unedited and edited sample genomic DNAs were extracted and edited fragments were amplified and subjected to Sanger sequencing. Editing efficacy was calculated by ICE analysis (https://www.synthego.com/guide/how-to-use-crispr/ice-analysis-guide/ accessed on 11 October 2023).

For protein level knock-out validation, 72 h after electroporation, BMDCs were treated for 4 h under hypoxia. Immunocytochemistry/immunofluorescence was performed on BMDCs cultured in 24-well polymer-treated plates (Cellvis P24) using standard indirect immunofluorescence procedures adapted from a macrophage staining protocol [20]. After hypoxia treatment, cells were washed with PBS, fixed in 4% PFA for 15–20 min at room temperature, and washed three times with PBS. Cells were then blocked and permeabilized in serum-containing buffer with 0.1% Triton X-100 for 30 min. Cells were incubated with primary anti-HIF1α antibody (Abcam, Waltham, MA, USA, cat# ab216842) overnight at 4 °C, followed by PBS washes and incubation with goat anti-rabbit Alexa Fluor 488 secondary antibody (Thermo Fisher Scientific, A-11008) at room temperature for an hour protected from light. Nuclei were counterstained with NucSpot 750/780 (Biotium, Fremont, CA, USA, cat# 41038). After final washes, cells were imaged on the Leica Stellaris confocal microscope. Cells were quantified and images were generated using Imaris post-processing software (Imaris 10.2.0).

### 2.10. Brain Slicing Culture

On the day of sectioning, naïve mice brain, primary tumor-bearing brain, and ACT-escaped tumor-bearing brain were carefully dissected and embedded in 4% ultra-low melting agarose (Sigma-Aldrich, Burlington, MA, USA, cat# A2576) in a specimen tube. The specimen tube was rapidly cooled with a chilling block and then inserted into the vibratome (VF-300, Precisionary, Ashland, MA, USA) in contact with a buffer tray filled with ice-cold dissection buffer. Sectioning was completed with settings of 2 mm/s speed, 5 Hz frequency, and 250 µm thickness. The brain slices were collected carefully to prevent damage to the tissue and placed onto a 0.4 µm pore 12-well plate cell insert (Thermo Fisher Scientific, cat. 140652) with 500 µL of pre-warmed brain slice culture medium (basal medium eagle [Thermo Fisher Scientific], 26.6 mM Hepes [pH 7.1; Thermo Fisher Scientific], 511 µM ascorbic acid, 1% [*v*/*v*] GlutaMAX [Thermo Fisher Scientific], 0.033% [*v*/*v*] insulin [Sigma-Aldrich], 1% [*v*/*v*] penicillin/streptomycin [Thermo Fisher Scientific], and 25% [*v*/*v*] heat-inactivated horse serum [Sigma-Aldrich]). Brain slices were placed in a CO_2_ incubator for 4 h before spent media was replaced with fresh medium. Continued culture was maintained for two days, after which the conditioned medium was collected for BMDC treatment.

### 2.11. Tissue Processing and Flowcytometry

Harvested tumors were chopped into small pieces of 2–4 mm and dissociated into single cell suspension by tumor dissociation enzyme mix (1 mg/mL collagenase IV, 100 U/mL Hyaluronidase, 15 U/mL DNase I in DMEM basal medium) in gentleMACS Octo Dissociator with Heaters (program 37C-m-TDK_2). Cells were filtered through 70 µm cell strainer. Debris was removed by debris removal kit (Mitenyi Biotec, Gaithersburg, MD, USA, cat# 130-109-398). For tumor-draining lymph node (tdLN), lymph nodes were harvested and minced in a 70 µm cell strainer. Cells went through strainer were collected. Count cell numbers and adjust cell density to around 10 million/mL in MACS buffer (2% FBS, 2 mM EDTA PBS). For flowcytometry, 100 ul cell suspension was used for staining. Cells were stained by viability dye (1:1000) for 10 min before FcX blocking (Biolegend, San Diego, CA, USA, cat# 101320) and monocyte blocking (Biolegend, San Diego, CA, USA, cat# 426103). Antibody mix was added after Fc and monocyte blocking and incubated for 15 min at 4 °C. Wash once by MACS buffer and fix cells in 1% PFA for 30 min at room temperature. The samples were run on flowcytometer (Symphony A3, BD biosciences, San Jose, CA, USA). Data was analyzed by Flowjo software (10.8.0). The gating strategy is shown in Supplementary Data (Figure S4). Antibodies used in phenotyping are listed in Supplemental Table S1.

### 2.12. T Cell Activation Assay

Dendritic cells sorted from tumor or spleen or BMDCs electroporated with total tumor RNAs were co-cultured with tumor-reactive T cells generated as described above at ratio of 1:10 (DC:T cell). Tumor-associated dendritic cells were FACS-sorted from pooled tumor samples obtained from 3 to 4 mice per repeat. Three repeats were generated for the co-culture experiments. T cell preparations quality was verified by measuring their proliferative response after restimulation; in all successful cultures, viability exceeded 85%. Tumor-reactive T cells were stained by celltrace violet (Thermo Fisher Scientific, C34557) before adding into co-culture. 48 h later, harvest co-culture supernatant for IFNgamma ELISA (R&D Systems, Minneapolis, MN, USA, cat# DY485-05) and harvest cells for Flowcytometry. Antibodies used for T cell proliferation and activation phenotyping are listed in Supplemental Tables S2 and S3.

### 2.13. Western Blot

BMDCs were treated by tumor brain slice culture conditioned medium (half conditioned medium and half DC culture medium) for 24 h. Cells pellets were lysed in lysis buffer with proteinase inhibitor. Vortex for 30 s and incubate in ice for 30 min. Centrifuge at 14,000× *g* for 10 min at 4 °C. Collect supernatant and determine protein concentration by BCA protein assay (Thermo Fisher Scientific, 23227). Add Laemmli sample buffer (Bio-Rad, 1610747) and denature at 95 °C for 10 min. An amount of 30 µg protein was loaded into 4–15% TGX precast protein Gels (Bio-Rad, 4561083) run in Tris-Glycine-SDS buffer. Proteins were transferred to PVDF membrane using semi-transfer system (Thermo Fisher Scientific, iblot2). Membrane was blocked in EveryBlot Blocking buffer (Bio-Rad, 12010020) for 5 min at room temperature. Protein blot was incubated in anti-HIF1α antibody solution (1:1000, Cell Signaling technology, Danvers, MA, USA, cat# 36169, RRID:AB_2799095) for overnight at 4 °C. Incubate blot in secondary antibody solution (1:5000, HRP-anti-rabbit IgG, Cell Signaling, 7074, RRID:AB_2099233) at room temperature for 1 h. After 3 times washing, detect HIF1α signal by incubating blot in chemiluminescent substrate solution and capture signal by charge-coupled device imagers. For blotting actin signal, blot was stripped by stripping buffer (Thermo Fisher Scientific, 21059) and repeating blocking and antibody incubation (beta-actin antibody, Cell Signaling, 8457, RRID:AB_10950489).

### 2.14. Statistical Analysis

Statistical tests were performed using GraphPad Prism 10. For in vitro experiments, we utilized the unpaired two-tail Student’s *t* test (two groups) or one-way ANOVA (more than two groups). Pearson correlation coefficient and *p* value were calculated in R using stat_cor() function for correlation plot.

### 2.15. Animal Study Design

For experiments that involve animal use, 10 mice were used for each treatment group (primary, ACT escaped). Tumor cells were implanted 2 weeks later in primary group mice than the ACT-treated group due to the different survival length according to the previous study. Tissue harvest was done on the same day when the animals were reaching endpoint (tumor size ≥ 0.8 cm). The number of animals used for tissue harvest for each group could be different as the number of animals meeting harvest criteria could be different in different groups. The actual animal size used for each experiment is indicated in the figure legend. Randomization was done after tumor implantation to allocate different treatment groups. No blinding was done while conducting the experiment as we needed to give treatment at different times. Immunophenotyping was assessed by flowcytometry from the tissue collected. Sample size is calculated using the pwr.anova.test() function in R with parameters set as follows: significance level = 0.05, power = 0.8, control mean = 5%, group mean = 10%, standard deviation = 3%.

## 3. Results

### 3.1. Adoptive Transferred T Cells Retained Non-Exhaustion and Cytotoxic T Cell Markers in ACT-Escaped Tumors

Our previous studies have shown that our ACT significantly increased the survival rate in adult and pediatric brain tumor mouse models [19,21,22]. Mechanistically, ACT treatment significantly increases DC infiltration into tumors, facilitating T cell-mediated anti-tumor immune responses [22]. However, even in mice that responded to ACT, most still eventually reached a humane endpoint due to tumor recurrence. Therefore, we asked why ACT treatment fails to provide durable long-term survival in these mice and what the mechanism is underlying the loss of a sustained antitumor response.

**Figure 1:**
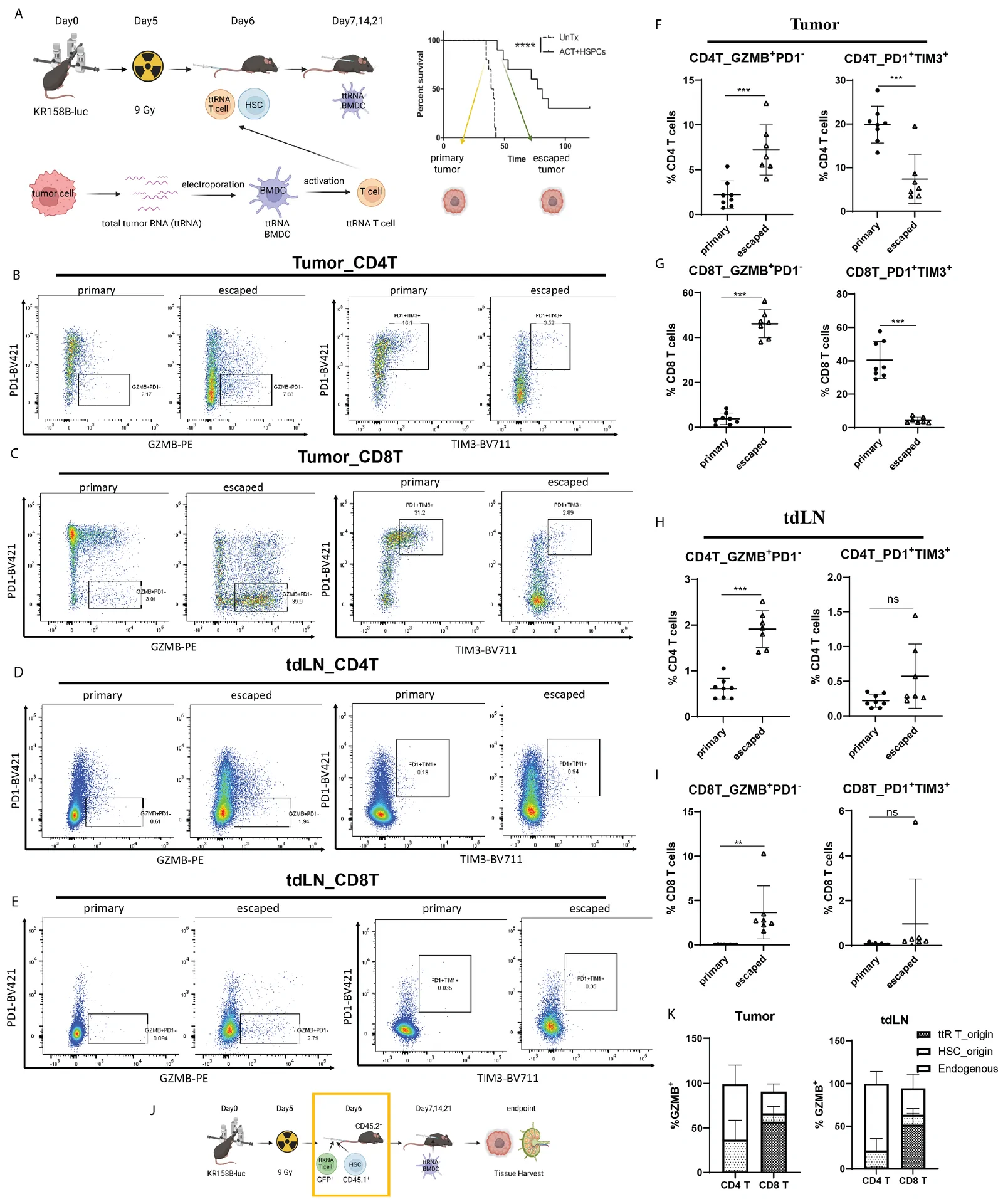
Adoptive transferred T cells retained non-exhaustion and cytotoxic T cell markers in ACT escaped tumors. (**A**) Illustration of ACT experimental design, primary and escaped tumors. ACT treatment starts 5 days after tumor implantation, 9 Gy whole-body irradiation was applied followed by HSC and tumor-reactive T cell transfer, and 3 doses of DC vaccines against primary tumor antigens. Tumor-reactive T cells were generated by priming mice with total KR158B-luc tumor RNA-pulsed BMDCs. Splenocytes from primed mice were restimulated by total tumor RNA-pulsed BMDCs. T cells expanded from the restimulation were used for ACT. Primary tumors were harvested in endpoint reaching KR158B-luc-bearing mice with no treatment. Escaped tumors were harvested in endpoint reaching KR158B-luc-bearing mice with ACT treatment. (**B**,**C**) Representative flowcytometry plots for GZMB^+^PD1^−^ and PD1^+^TIM3^+^ CD4 T cell and GZMB^+^PD1^−^ and PD1^+^TIM3^+^ CD8 T cell gatings in primary and escaped tumors. (**D**,**E**) Representative flowcytometry plots for GZMB^+^PD1^−^ and PD1^+^TIM3^+^ CD4 T cell and GZMB^+^PD1^−^ and PD1^+^TIM3^+^ CD8 T cell gatings in tdLNs from primary and escaped mice. (**F**,**G**) Quantitative data for (**B**,**C**). (**H**,**I**) Quantitative data for (**D**,**E**). (**J**) Illustration of experimental design for tracing the source of T cells in ACT-escaped tumor and tdLN. Adoptive transferred T cells (ttR-T) were labeled with GFP, HSCs were labeled with CD45.1, endogenous T cells were labeled with CD45.2. Tumors and tdLNs were harvested from ACT-escaped mice for flowcytometry. (**K**) Ratios of different origin T cells in GZMB^+^ CD4 and CD8 T cells in tumors and tdLNs were determined by flowcytometry. *p* values < 0.01, 0.001, 0.0001 were indicated as “**”, “***”, “****”, respectively.

T cell exhaustion is considered a major factor contributing to resistance to cancer immunotherapy, as consistent exposure to tumor antigens can induce T cell exhaustion in the tumor microenvironment [23]. We are wondering whether T cell exhaustion is involved in the immune escape in our ACT platform. To address this question, we evaluated the cytotoxicity and exhaustion markers on the T cells in ACT-escaped tumor and primary tumor under no treatment. KR158B-luc-implanted mice were treated with ACT including whole-body irradiation followed by HSC and tumor-reactive T cell transfer, and 3 doses of DC vaccines against primary tumor antigens as illustrated in Figure 1A. Based on the work by Drs. Rosenberg, Restifo, and Dudley at the NCI and our previous work, whole-body myeloablation before cellular transfer enhances adoptive cell therapy (ACT) efficacy [24,25,26,27]. Mice that developed recurrent tumors exceeding 0.5 cm were subjected to tissue harvest for collection of ACT-escaped tumors or tumor-draining lymph nodes (tdLNs), as illustrated in Figure 1A. Primary tumors and tdLNs were harvested from untreated mice bearing tumors larger than 0.5 cm as illustrated in Figure 1A. Surprisingly, we found that the GZMB^+^PD1^−^ population of CD8^+^ and CD4^+^ T cells was even higher in ACT-escaped tumors compared to primary tumors (Figure 1B,C,F,G), and similar trends were observed in tdLNs (Figure 1D,E,H,I). We also found no increase in exhausted T cells (TIM3^+^PD1^+^) in ACT-escaped tumors compared to primary tumors (Figure 1B,C,F,G). In fact, there was a decrease in T cell exhaustion in ACT-escaped tumors compared to primary tumors (Figure 1B,C,F,G). No difference in T cell exhaustion was seen in the tdLNs (Figure 1D,E,H,I). These results suggest that unlike primary tumors, intratumoral T cell exhaustion or loss of cytotoxicity may not be the main driver during immune escape in ACT-escaped tumors.

The question remains as to why these cytotoxic T cells are not attacking tumor cells. As previously mentioned, tumor antigen shifting is one of the mechanisms in ACT-escaped tumors, where T cells generated against primary tumor antigens are unable to recognize the escaped tumor cells and vice versa. Only T cells generated against ACT-escaped tumor antigens can effectively recognize and kill the ACT-escaped tumor cells [16]. We hypothesized that these cytotoxic T cells are derived from adoptively transferred T cells, which were generated against primary tumor antigens and, therefore, fail to recognize newly evolved tumor antigens in ACT-escaped tumors. To test this hypothesis, we labeled different sources of T cells in ACT treatment with distinct markers: GFP for adoptively transferred T cells, CD45.1 for hematopoietic stem cell (HSC)-derived T cells, and CD45.2 for endogenous T cells. Tumors and tdLNs were harvested at the endpoint to determine the origin of the GZMB^+^ T cells in ACT-escaped mice (Figure 1J). Our results showed that the majority of GZMB^+^ CD8 T cells were derived from adoptively transferred T cells in both tumors and tdLNs (Figure 1K). In contrast, the majority of GZMB^+^ CD4 T cells were derived from endogenous T cells, with barely any GZMB^+^ CD4 T cells coming from adoptively transferred T cells (Figure 1K).

In summary, these results suggest that though the persistence of GZMB-expressing and non-exhausted characteristics in adoptively transferred T cells in ACT-escaped tumors, they failed to recognize the escaped tumor antigens possibly due to antigen shifting in ACT-escaped tumor.

### 3.2. ACT-Escaped Tumor-Associated DCs Display Impaired T Cell Activation

Dendritic cells are crucial in initiating and sustaining anti-tumor response by continuously processing neoantigens in tumor site and eliciting a new spectrum of T cells in lymphoid organs. Our previous studies have shown that ACT treatment significantly increases DC infiltration into tumors, facilitating T cell-mediated anti-tumor immune responses [22]. Given the critical role of DCs in immune activation against tumors during ACT, we asked whether DCs in ACT-escaped tumors are functionally impaired.

**Figure 2:**
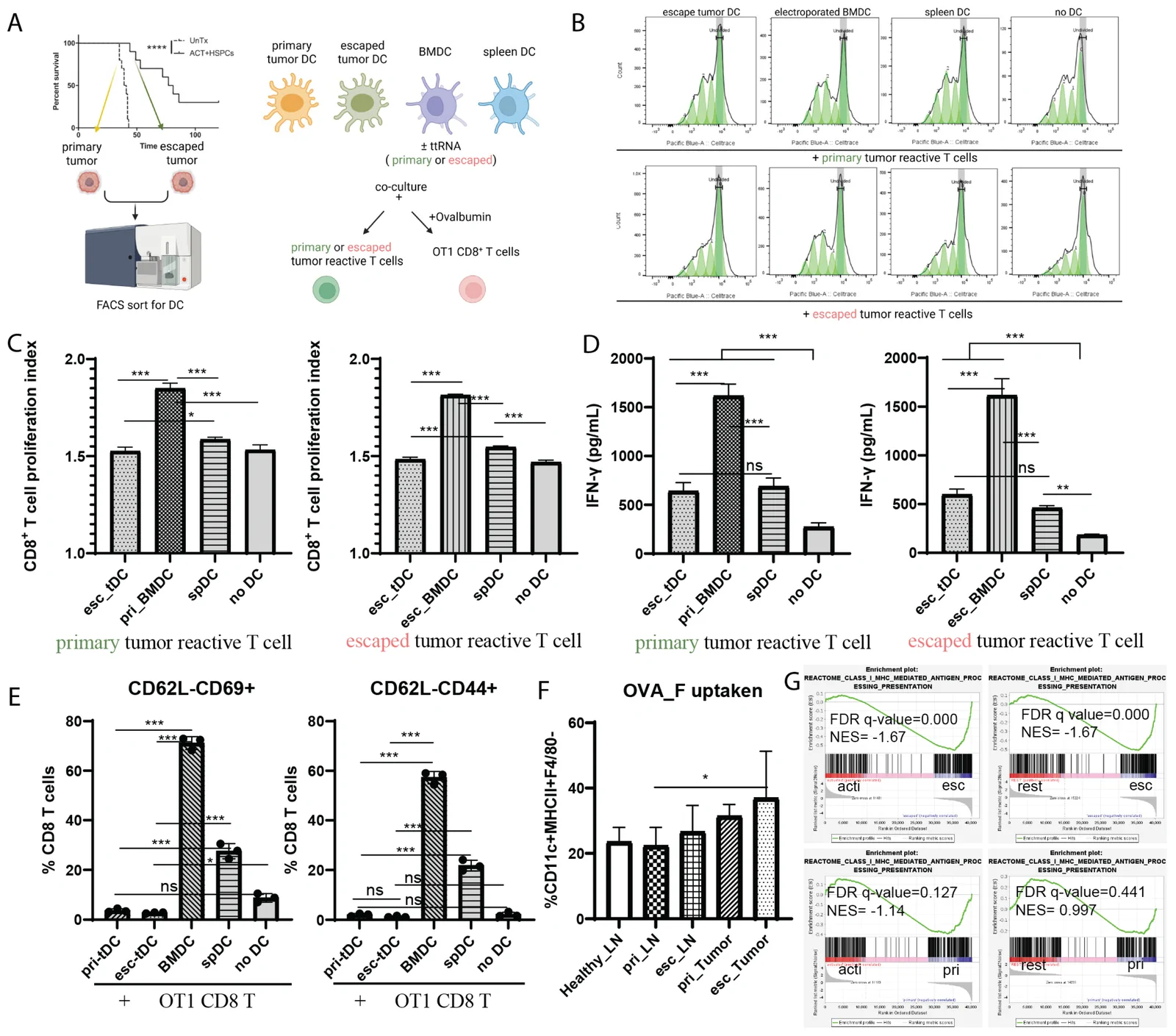
ACT-escaped tumor-associated DCs are impaired in T cell activation. (**A**) Illustration of tumor-associated DC isolation and experimental design for DC functional assay (Figure 2B–E). Primary and escaped tumors were obtained from endpoint tumor-bearing mice and DCs were FACS isolated by CD45^+^MHCII^+^CD11c^+^ markers. Primary tumor DCs, escaped tumor DCs, BMDCs electroporated with total tumor RNA from primary or escaped tumor, spleen DCs were subjected to co-culture experiments with primary tumor-reactive T cells or escaped tumor-reactive T cells. And co-culture with OT CD8 T cells with the addition of ovalbumin. (**B**) Celltrace signal indicating T cell proliferation in co-culture assay with tumor-reactive T cells. pri-tDC: primary tumor DC, esc-tDC: escaped tumor DC, pri_BMDC: BMDC electroporated with primary tumor RNA, esc_BMDC: BMDC electroporated with escaped tumor RNA, spDC: spleen DC from naïve mice. (**C**) Quantitative summary of T cell proliferation index in (Figure 2B). (**D**) IFNγ secretion in tumor-reactive T cells co-culture assay groups. (**E**) T cell activation determined by CD62L-CD69^+^ and CD62L-CD44^+^ markers in OT1 CD8 T co-culture assay. Non-electroporated BMDC and spDC were used as positive controls. (**F**) Antigen uptake capability in DCs from different group tissue was determined by fluorescein-conjugated ovalbumin signal. Cells were subjected for flowcytometry after 30 min culture in the presence of fluorescein-conjugated ovalbumin. (**G**) Gene signature enrichment analysis of MHC-I antigen presentation genes in primary and escaped tumor DCs. Rest: resting spleen DCs isolated from healthy mice; Acti: spleen DCs isolated from LPS and ovalbumin active mice; Pri: primary tumor DCs isolated from KR158B-luc tumor with no treatment (as indicated in Figure 2A); Esc: escaped tumor DCs isolated from KR158B-luc tumor escaped from ACT treatment (as indicated in Figure 2A). FDR-q value < 0.25 is considered significant. The mean  ±  SD is indicated. One way ANOVA (more than two groups) was performed by Graphpad Prism 10.0 for (**C**–**F**). *n* = 3; *p* values < 0.05, 0.01, 0.001, 0.0001 were indicated as “*”, “**”, “***”, “****”, respectively.

To address this question, we assessed T cell activation as a proxy for DC function (Figure 2A). DCs sorted by fluorescence-activated cell sorting (FACS) from ACT-escaped tumors were co-cultured with tumor-reactive T cells. Splenic DCs (spDCs) from naïve mice served as negative controls, as they had not been exposed to tumor antigens, so they are not expected to activate tumor-reactive T cells. BMDCs electroporated with primary or escaped tumor RNAs served as positive controls, which will present tumor antigens and activate its respective tumor-reactive T cells. Tumor-reactive T cells against either primary tumors or escaped tumors were generated via T cell stimulation by primary or escaped tumor RNA-loaded DCs respectively, as previously described [21]. Results showed that T cell proliferation rates, determined by CellTrace dye, in the escaped tumor DC co-culture group had no significant difference compared to negative controls and were significantly lower than positive controls in both co-cultures with primary and escaped tumor-reactive T cells (Figure 2B,C). IFN-γ secretion also showed consistent results, with no significant differences between escaped tumor DC co-cultures and negative controls (Figure 2D). These results suggested that DCs in ACT-escaped tumors are disabled in activating T cells.

DC dysfunction in tumors has been reported [4,28]. We wondered whether there were any functional differences between DCs from primary and ACT-escaped tumors. Results showed that both DCs from primary and ACT-escaped tumors were deficient in activating tumor-reactive T cells (Figure S1A–C). In the co-culture with tumor-reactive T cells, we did not provide an external antigen in the culture, which meant we could not determine whether the failure in T cell activation was due to DC dysfunction or the lack of accessible tumor antigens. Given this consideration, we assessed DC function by providing the antigen in the culture. We co-cultured DCs from both primary and escaped tumors with OT1 CD8^+^ T cells in the presence of ovalbumin (Figure 2A). Results showed that both primary and escaped tumor DCs failed to further activate OT1 CD8^+^ T cells compared to BMDCs and splenic DC positive controls, and there was no significant difference between primary and escaped tumor DCs (Figure 2E). Overall, these results suggest that DCs from both primary and ACT-escaped tumors are dysfunctional in activating T cells.

Given that DC-mediated T cell activation was impaired, we next wanted to determine if this failure was a result of dysfunctional phagocytosis. Antigen-presenting cells utilize phagocytosis to uptake antigens. To evaluate phagocytosis, we incubated DCs with fluorescence-conjugated ovalbumin. As shown in Figure 2F, 30% of DCs from both primary and ACT-escaped tumors exhibited positive antigen uptake signals after 30 min of incubation with fluorescence-conjugated ovalbumin, which was even slightly higher than the uptake rate observed in positive control DCs from healthy lymph nodes (Figure 2F). In addition, a similar uptake rate was observed in DCs from tumor-draining lymph nodes (tdLNs) and healthy LNs, indicating that there was no difference between these groups (Figure 2F). This result suggests that the failure of T cell activation by tumor-associated DCs is not due to impairment of phagocytosis. Instead, this finding suggests that the dysfunction of tumor-associated DCs may be related to antigen processing and presentation, rather than phagocytosis of antigens.

To further investigate the mechanism of DC dysfunction in primary and ACT-escaped tumors, we conducted bulk RNA sequencing on DCs FACS-sorted from both tumors, along with resting status splenic DCs from naïve mice and activated status splenic DCs from mice treated with lipopolysaccharide (LPS) and ovalbumin. We found that DCs from escaped tumors exhibit a negative enrichment of MHC-I antigen-presentation genes, no matter compared with splenic activated or resting DCs, suggesting an impaired MHC-I antigen presentation in ACT-escaped tumor-associated DCs (Figure 2G). In contrast, MHC-II antigen presentation genes are not downregulated in ACT-escaped tumor DCs (Figure S1D), suggesting MHC-I antigen presentation dysregulation may lead to the dysfunction of ACT-escaped tumor DCs. In primary tumor DCs, MHC-I antigen presentation genes are negatively enriched only when compared with splenic activated DCs, but show no significant enrichment versus splenic resting DCs (Figure 2G). However, MHC-II antigen presentation genes are significantly negatively enriched in primary tumor DCs, compared with either splenic activated or resting DCs, suggesting a discrepancy of mechanisms in driving DC dysfunction in primary and escaped tumor DCs (Figure S1D). Overall, consistent with the functional assay, these results suggest that tumor-associated DCs in both primary and ACT-escaped tumors are impaired in antigen presentation and the underlying mechanisms could be different.

### 3.3. An Enhanced Tolerance in ACT-Escaped Tumor-Associated DCs Regulated by Hypoxia Pathway

**Figure 3:**
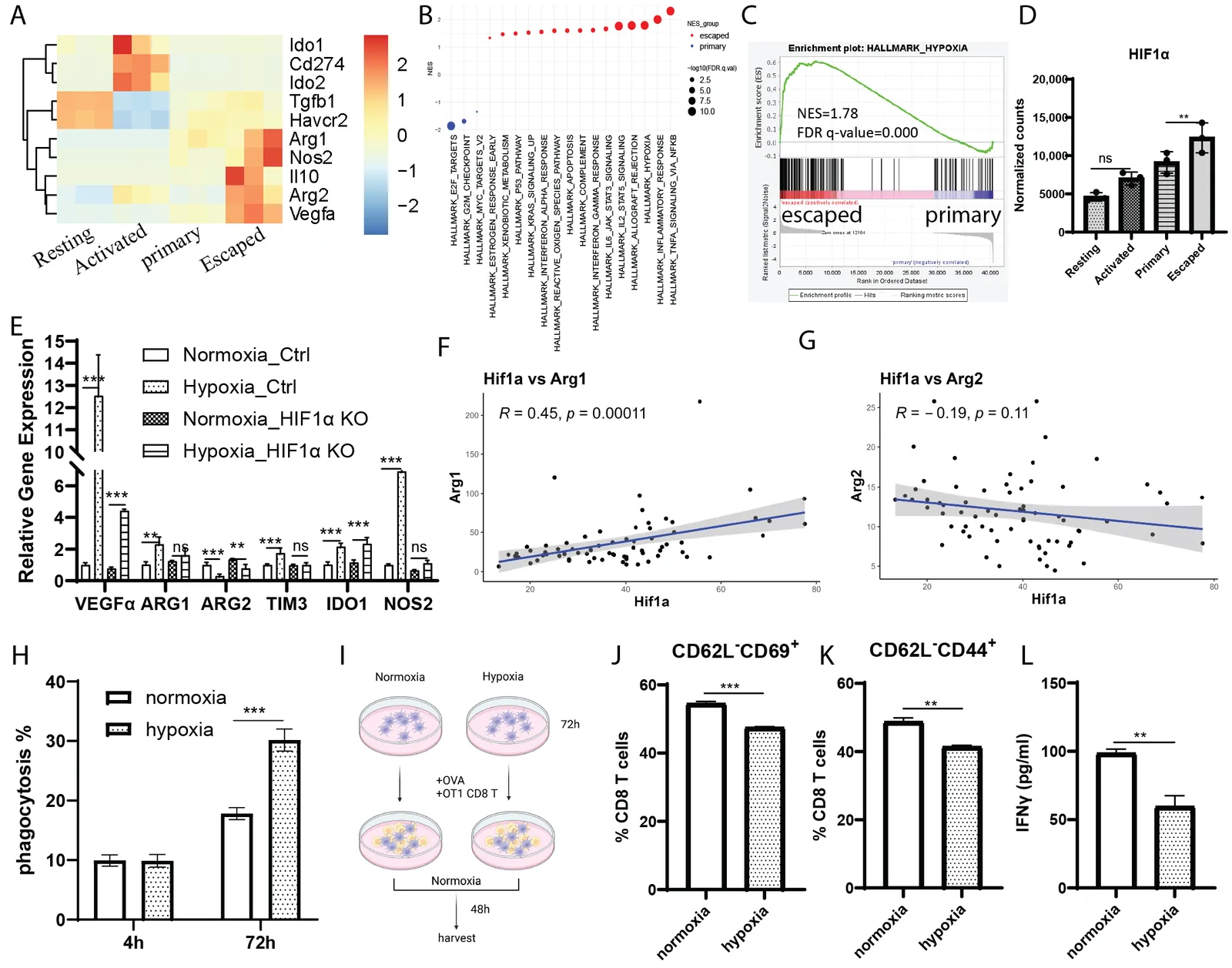
An enhanced tolerance in ACT-escaped tumor-associated DCs regulated by hypoxia pathway. (**A**) Heatmap of DC tolerance-associated gene expression. Resting: resting spleen DCs isolated from healthy mice; Activated: spleen DCs isolated from LPS and ovalbumin active mice; Primary: primary tumor DCs isolated from KR158B-luc tumor with no treatment (as indicated in Figure 1A); Escaped: escaped tumor DCs isolated from KR158B-luc tumor escaped from ACT treatment (as indicated in Figure 1A). (**B**) GSEA for pathways enriched in primary tumor DCs and escaped tumor DCs. Only pathways with FDR q value < 0.1 are shown. (**C**) GSEA plot for hypoxia pathway. (**D**) HIF1α gene expression level from RNA seq data. (**E**) Gene expression level by RT-qPCR in control and HIF1α knock-out BMDCs under normoxia and hypoxia. (**F**,**G**) Correlation plot for HIF1α and Arg1, HIF1α and Arg2 gene expression in different regions of KR158B-luc tumor brain. Each dot represents the gene expression from one selected region in spatial transcriptomics data. Correlation coefficient and *p* value were calculated by the Pearson method. (**H**) Phagocytosis rate of BMDCs under normoxia and hypoxia condition determined by the uptake of fluorescein-conjugated OVA. (**I**) Illustration for assessing T cell activation function by DCs treated by normoxia or hypoxia. BMDCs were treated under normoxia and hypoxia for 72 h, then were harvested for co-culture with OTI CD8 T under normoxia condition for 48 h in the presence of ovalbumin. (**J**) T cell activation marker CD62L^−^CD69^+^ was determined by flowcytometry for T cells harvested from experiment Figure 3I. (**K**) T cell activation marker CD62L-CD44+ was determined by flowcytometry for T cells harvested from experiment Figure 3I. (**L**) IFNγ secretion in DC-T co-culture from experiment Figure 3I was determined by ELISA. The mean  ±  SD is indicated. One way ANOVA (more than two groups) was performed by Graphpad Prism 10.0 for (**D**,**E**). Significance in (**H**,**J**–**L**) was calculated using a two-sided unpaired *t*-test. *n* = 3; *p* values < 0.01, 0.001 were indicated as “**”, “***” respectively. Data were collected from 3 independent repeats.

DC tolerization has been demonstrated as a mechanism of tumor-induced DC dysfunction [29]. Given this context, we compared the gene expression levels of DC tolerization genes in our samples. Notably, we found a higher level of Arg1, Arg2, Nos2, IL10, and TGFβ in ACT-escaped tumor DCs compared with the other three groups (Figure 3A). This suggests an enhanced tolerance in DCs in ACT-escaped tumor compared to primary tumor. Interestingly, IDO1/2 and PDL1 were significantly increased in activated splenic DCs compared with resting status splenic DCs, but not in tumor-associated DCs (Figure 3A). This indicates that there is an early rise in DC tolerization after DC activation, which is distinct from the one driven by tumor. To investigate the upstream pathway that induces the tolerance in ACT-escaped tumor DCs, we analyzed the pathways enriched from RNA sequence data. Gene set enrichment analysis (GSEA) revealed that the hypoxia signaling pathway is significantly enriched in escaped tumor DCs compared to primary tumor DCs or splenic DCs in activated and resting states (Figure 3B,C and Figure S2C–G). HIF1a, which is a transcription factor that drives hypoxia pathway activation, has the highest expression level in ACT-escaped DCs among the four groups (Figure 3D). We also noticed that the most suppressed pathways in ACT-escaped tumor DCs compared with primary tumor DCs are cell cycle-related pathways, E2F targets and G2M checkpoint (Figure 3B). Consistent with this result, we detected reduced DC subpopulations in CD45^+^ population in escaped tumor rather than primary tumor, including pDC, moDC, cDC1, cDC2 (Figure S2A,B). These results suggest an intrinsic defect in the maintenance of DC function and populations in ACT-escaped tumor compared with primary tumor.

It has been reported that hypoxia induces Arg1 expression [30], which prompted us to further investigate the relationship between hypoxia and the DC tolerization program induced in ACT-escaped tumor DCs. We first assessed whether hypoxia can induce DC tolerance gene expression. Comparing gene expression in DCs cultured under hypoxia and normoxia, we found that multiple DC tolerance-associated genes, including *ARG1*, *TIM3*, *IDO1*, *NOS2*, but not *ARG2* were induced by hypoxia (Figure 3E). We used Cas9 CRISPR to knock-out HIF1α gene in BMDCs by electroporation, which achieved 56% editing efficacy in both guide RNAs and validated by immunofluorescence at protein level (Figure S2H,I). Despite only about a 50% knock-out of HIF-1α in BMDCs, we still observed a dramatic abolishment in the induction of DC tolerance genes, including *ARG1*, *TIM3*, and *NOS2*, from hypoxia treatment (Figure 3E and Figure S2J), suggesting HIF-1α mainly regulates these gene expressions. However, we also observed that some other DC tolerance genes, *ARG2* and *IDO1*, are not regulated by HIF-1α. Though induced by hypoxia, this induction of *IDO1* expression is independent of the *HIF1α* gene. Unlike *ARG1*, *ARG2* expression is even suppressed by hypoxia, and knocking out *HIF1α* released the suppression (Figure 3E and Figure S2J). The induction of the classic hypoxia-response gene *VEGFα* was also significantly attenuated by *HIF-1α* knock-out (12.5-fold increase in wild type BMDCs versus 4.3-fold increase in *HIF1-1α* knock-out) (Figure 3E and Figure S2J). The incomplete abolishment is possibly due to the half knock-out. Overall, these results suggest that the hypoxia pathway induces DC tolerance genes, and HIF-1α is one of the regulators of the induction.

Tumors exhibit regions with varying levels of oxygen, leading to the intratumoral uneven activation of hypoxia pathways. To validate the existence of hypoxia pathway regulation of tolerance genes in tumor mass, we analyzed the correlation of gene expression levels of HIF1α and ARG1 in different regions of the tumor using spatial transcriptomics. The data revealed a significant correlation between *HIF1α* and *ARG1*, but not with *ARG2* (Figure 3F,G). These findings demonstrate that the hypoxia pathway regulates tolerance-related genes in DCs and mainly dependent on HIF1α, with *ARG2* expression being negatively regulated by the hypoxia pathway.

We then assessed whether hypoxia would attenuate dendritic cell function. We found that hypoxia treatment even enhanced phagocytosis capability of dendritic cells after 72 h of hypoxia treatment (Figure 3H). No difference was observed after 4 h of hypoxia treatment. This finding is consistent with the report that hypoxia increases phagocytosis in macrophages [31]. We further assessed the T cell activation capability by co-culturing hypoxia-treated DCs with OT1 CD8 T cells in the presence of ovalbumin (Figure 3I). Notably, we found that DCs treated under hypoxia showed less T cell activation markers (CD62L^−^CD69^+^, CD62L^−^CD44^+^) and less IFNγ secretion compared to the ones treated under normoxia (Figure 3J–L). These results showed that hypoxia impairs DC function in T cell activation, which is downstream of phagocytosis, consistent with a role in suppressing antigen presentation through tolerization.

IL10 and TGFβ induce Tregs [32]. Do the increased IL10 and TGFβ in ACT-escaped tumor-associated DCs induce more Tregs? Interestingly, although GZMB^+^PD1^−^ CD4 T cells comprised only 5% of the total CD4 T population in the tumor and even less in the tdLN (Figure 1B,D,F,H), we found that 80% of these GZMB^+^PD1^−^ CD4 T cells were FoxP3 positive, a regulatory T cell (Treg) marker, in both primary and escaped tumors and tdLNs (Figure S3A,B). Studies have shown that GZMB-expressing Tregs promote tumor progression and that GZMB from Tregs is crucial for its inhibition of tumor clearance [33,34]. Although we did not observe an increased accumulation of Tregs (FoxP3^+^ CD4^+^ T cells) in the escaped tumor compared to the primary tumor, we did find an increase in Tregs in the tdLNs from ACT-escaped tumor-bearing mice (Figure S3C,D). However, hypoxia treated DC conditioned medium does not induce Tregs in vitro (Figure S3E), suggesting that a more complex Treg inducing mechanism in vivo.

### 3.4. Immune Cell Infiltration and Inflammation Enhanced Hypoxia Signaling Activation and Tolerance of DCs in ACT-Escaped Tumor

**Figure 4:**
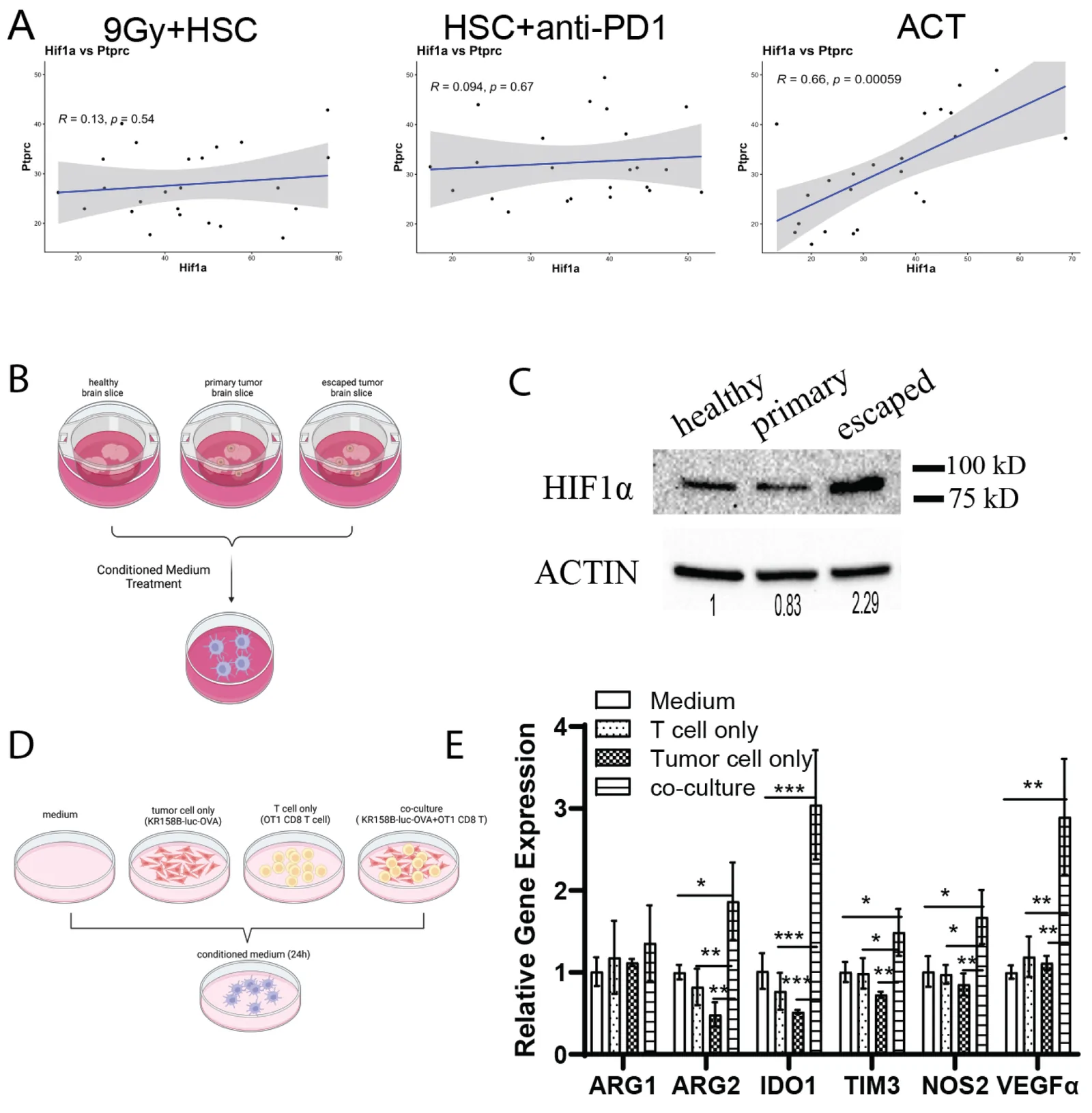
Immune cell infiltration and Inflammation enhanced hypoxia signaling activation and tolerance of DCs in ACT-escaped tumor. (**A**) Correlation plot for HIF1α and Ptprc (CD45) gene expression in different regions of KR158B-luc tumor brain from 9 Gy + HSC, HSC + αPD1, and ACT groups. Each dot represents the gene expression from one selected region in spatial transcriptomics data. Correlation coefficient and *p* value were calculated by the Pearson method. (**B**) Illustration for BMDCs treated by brain slice conditioned medium. (**C**) HIF1α protein levels in BMDCs treated by conditioned medium from different brain slice culture as indicated in Figure 4B were determined by Western blot. Relative expression of HIF1α to ACTIN is indicated by number. (**D**) Illustration of BMDCs treated by conditioned medium from KR158B-luc-OVA and OTI CD8 T cell culture. (**E**) Gene expression in BMDCs treated with different conditioned medium as indicated in Figure 4D was determined by RT-qPCR. The mean  ±  SD is indicated. One way ANOVA (more than two groups) was performed by Graphpad Prism 10.0 for Figure 4E. *n* = 3; *p* values < 0.05, 0.01, 0.001 were indicated as “*”, “**”, “***” respectively. Data was collected from 3 independent biological repeats. The original blots can be found in Supplementary File S1.

Hypoxia is a characteristic feature of the tumor environment, but why does ACT lead to a higher level of hypoxia pathway activation? Hypoxia and inflammation frequently co-exist as microenvironmental features at sites of concentrated physiological or pathological immune activity [10]. The highly proliferative infiltration of immune cells into tumors could potentially burden the oxygen supply, thereby leading to enhanced hypoxia. Consistent with this hypothesis, we found that CD45 expression, as an indication of immune cell population, has a significant positive correlation with HIF1α expression in spatial transcriptomic data from tumor-bearing brain slices after ACT treatment, but not in tumor brain slices from the 9 Gy irradiation + HSC group and HSC + anti-PD1 group (Figure 4A), suggesting that the transferred tumor-reactive T cells from ACT treatment may be associated with the correlation between CD45 and HIF1α in ACT-escaped tumor brain. The immune infiltration following ACT treatment could potentially induce higher levels of hypoxia in the tumor microenvironment. We also found a significant correlation between the proliferation marker Ki67 and HIF1α (R = 0.35, *p* = 0.0027) when the brain slices from all three treatment groups were combined analyzed (Figure S4A). Within each individual group, the correlation did not reach significance—likely because each group had a smaller sample size than the combined analysis. However, the ACT‑treated group showed the strongest (though still non‑significant) trend, with R = 0.38 (*p* = 0.071), compared to the HSC + anti‑PD1 group (R = 0.36, *p* = 0.093) and the 9 Gy + HSC group (R = 0.20, *p* = 0.36) (Figure S4A). These findings suggest that ACT‑mediated infiltration of proliferative immune cells may locally elevate hypoxic conditions within the tumor mass.

We further investigated whether the products generated in ACT-escaped tumor brain regulate the activation of the hypoxia pathway. Organotypic slice cultures have emerged as a promising model recapitulating precisely specific in vivo phenotypes [35]. We adopted this method to harvest conditioned medium from organotypic brain slice cultures from different brain groups, including healthy brain, primary tumor brain, and ACT-escaped tumor brain. We then treated DCs with these different conditioned media and detected HIF1α protein levels (Figure 4B). The results showed that conditioned medium from ACT-escaped brain cultures induced more HIF1α expression in DCs compared to the other groups (Figure 4C and Figure S4B). This suggests that there are factors secreted from ACT-escaped tumor brain that can induce higher hypoxia pathway activation.

Next, we investigated whether the secretions from T cell and tumor reactions can induce hypoxia pathway activation and thereby strengthen DC tolerance. We harvested conditioned medium from the co-culture of KR158B-luc-OVA cells and OT1 CD8 T cells, as well as from T cell and KR158B-luc-OVA cell cultures separately (Figure 4D). We then compared the expression of hypoxia response gene and DC tolerance genes in DCs treated with conditioned medium from these different groups (Figure 4E). Our results showed that VEGFα, a typical target of the hypoxia pathway, was distinctly induced in the group treated with co-culture conditioned medium. Similarly, DC tolerance genes IDO1, TIM3 and NOS2 are all distinctly induced by co-culture conditioned medium. Notably, ARG2 but not ARG1 expression was induced by co-culture conditioned medium (Figure 4E). These findings demonstrate that the secretions from T cell and tumor reactions, as another stimulus, can induce DC tolerance gene expression including ARG2, which is typically not induced by hypoxia. In line with the RNA sequence data, which showed that both ARG1 and ARG2 are induced at higher levels in ACT-escaped tumor DCs, it suggests that a combined effect from hypoxia and inflammation reactions enhances DC tolerance.

## 4. Discussion

In this study, we uncovered an additional layer of immune evasion mechanisms in ACT-escaped brain tumors, which involved DC dysfunction through hypoxia pathway-induced DC tolerance. This mechanism complements the previously reported tumor-antigen shifting and sheds light on why durable anti-tumor responses fail under ACT. We found that the adoptive transferred T cells still retain the cytotoxic and non-exhausted features in ACT-escaped tumors, suggesting that T cell dysfunction plays a less dominant role in this recurrence mechanism under ACT. Instead, we found DCs in ACT-escaped tumors are impaired in T cell activation. Further we found that an enhanced tolerance in ACT-escaped tumor-associated DCs regulated by the hypoxia pathway. The infiltration of immune cells and inflammation reactions triggered by ACT treatment enhanced tolerogenic features in DCs within ACT-escaped tumors. Tumors are inherently capable of evolving in response to treatment pressures. Our previous study showed the existence of tumor antigen shifting in ACT-escaped tumor and the adoptive transferred T cells against primary tumor antigens fail to recognize the shift tumor antigens in escaped tumor. Dendritic cells serve as sentinel cells, identifying neoantigens and orchestrating the activation of T cells to target evolved tumors. DC dysfunction will lead to failure in activating a new repertoire of T cells capable of recognizing these shifted tumor antigens, ultimately compromising a sustained antitumor response.

The findings of this study provide a compelling rationale for targeting DC dysfunction as a therapeutic strategy to achieve more sustained benefits. Future research should focus on determining whether inhibiting hypoxia pathway activation in disabled DCs would lead to prolonged survival benefits.

The relationship between hypoxia and inflammation and its role in inducing DC tolerance can be explained in two arms (as indicated in graphic abstract): (1) ACT brings more immune infiltration at the tumor site. The highly proliferative infiltration of immune cells into tumors could outstrip oxygen supply, elevating local hypoxia. Hypoxia, in turn, induces DC tolerance. (2) ACT also brings more inflammation reactions, and the products secreted from these reactions could induce DC tolerance genes as well, as indicated by Figure 4E. These two arms jointly promote DC tolerance: (1) Compensating in inducing tolerance genes. Inflammation reaction factors preferentially induce ARG2, whereas hypoxia induces ARG1. (2) Inducing more shared tolerance genes, TIM3, IDO1, and NOS2. Though we do see that VEGFα, a canonical target of the hypoxia pathway, is induced by inflammation reaction factors, suggesting inflammation reaction factors may induce hypoxia pathway activation, more direct evidence is needed to draw that conclusion. The coexistence of these two pathways synergistically elevates the expression of overlapping tolerance genes, thereby reinforcing DC tolerance. Whether inflammation directly activates hypoxic signaling remains to be confirmed; even if it does not, its independent regulation can still amplify the shared set of tolerance targets. Further mechanistic studies are needed to clarify the relationship between inflammation and hypoxia, and what secret factors are inducing DC tolerance genes, and what are the mediators in DCs connecting secreting factors with downstream DC tolerance genes.

The findings of this study also raise several intriguing questions for further investigation. Is there a quantitative impact of the hypoxia pathway activating signaling on DC function? As is known, hypoxia is a hallmark of solid tumors, regardless of treatment status. Our data indicate that DCs in ACT-escaped tumors exhibit an increased level of hypoxia pathway activation compared to primary tumors without treatment, in line with the development of DC tolerance. Notably, our RNA sequencing data (Figure 3A) show that primary tumor DCs already display a slightly increased expression of DC tolerogenic genes compared to splenic DCs, while ACT-escaped DCs exhibit a significantly higher level of DC tolerogenic gene expression. We wonder whether this difference is due to varying doses of hypoxia pathway activation stimuli or, alternatively, distinct downstream targets of different hypoxia pathway activation stimuli, such as hypoxia versus inflammation. While our data suggest that both mechanisms coexist, the infiltration of immune populations, which burdens the oxygen supply, leads to a higher intensity of hypoxia signals. The secretion of stimuli from T cell and tumor cell reactions targets different tolerance gene (ARG2) downstream of hypoxia stimuli. To further elucidate the mechanistic insight, evidence from additional studies is highly encouraged.

Although the current study primarily focuses on the intrinsic DC function impairment, our findings also suggest that there are interactive impacts in the tumor environment, including the promotion of an immunosuppressive microenvironment. Consistent with other studies, tolerogenic DCs may contribute to an immunosuppressive microenvironment by promoting the differentiation of Tregs [36]. In line with this, we observed higher levels of TGF-β and IL-10 expression in DCs from ACT-escaped tumors compared to primary tumors, which is known to promote Treg differentiation [37]. Furthermore, we found an increase in Tregs in tumor-draining lymph nodes (tdLNs) from ACT-escaped tumor-bearing mice compared to those bearing primary tumors. Our results support the hypothesis that tolerogenic DCs migrate to tdLNs and induce Tregs in these lymphoid organs, thereby contributing to immune escape. The mechanism we have identified may be shared with other immunotherapies and provides insights into understanding the immune escape alone with immunotherapies.

Future studies should explore whether launching a long-term antitumor immunity can be achieved by disrupting the hypoxia–tolerogenic axis in combination with different immunotherapies including but not limited to ACT. Future study should also focus on identifying the secreted factors from immune–tumor reactions that mediate DC tolerance, which will provide other therapeutic targets to achieve long-term benefits.

The study has limitations including reliance on a single KR158B-luc tumor model, Limited temporal analysis of immune cell dynamics during tumor progression and recurrence, lack of validation using human GBM samples, incomplete identification of the soluble factors responsible for DC tolerization.

## 5. Conclusions

In this study, we uncovered an additional layer of immune evasion mechanisms in ACT-escaped brain tumors, which involved DC dysfunction through hypoxia pathway-driven DC tolerance. This mechanism complements the previously reported tumor-antigen shifting and sheds light on why durable anti-tumor responses fail under ACT. The markedly increased infiltration of immune cells and the inflammation triggered by ACT treatment potentially amplify the hypoxia pathway and enhance DC tolerance in escaped tumor DCs. The impaired DC function leads to failure in activating a new repertoire of T cells capable of recognizing the shifted tumor antigens in ACT-escaped tumor. Thus, sustained anti-tumor immunity is compromised. Our study highlights the hypoxia–DC axis as a potential intervention target to enhance durable immunotherapy efficacy.

## Data Availability

All data relevant to the study are included in the article or uploaded as Supplementary Materials.

## Supplementary Materials

The following supporting information can be downloaded at: https://www.mdpi.com/article/10.3390/cancers18162669/s1, File S1: The original blots. Figure S1: (A) Illustration of experimental design for T cell activation assay (Figure S1B). (B) Fast proliferating CD8 T cells with low celltrace signal in co-culture with primary tumor reactive T cells were determined by flowcytometry. pri-tDC: primary tumor DC; pri_BMDC: BMDC electroporated with primary tumor RNA; spDC: spleen DC from naïve mice; no DC: no DCs were added. (C) Fast proliferating CD8 T cells with low celltrace signal in co-culture with escaped tumor reactive T cells were determined by flowcytometry. esc-tDC: escaped tumor DC; esc_BMDC: BMDC electroporated with escaped tumor RNA; spDC: spleen DC from naïve mice; no DC: no DCs were added. The mean ± SD is indicated. One way ANOVA (more than two groups) were performed by Graphpad Prism 10.0 for (B,C). *n* = 3, *p* value < 0.01, 0.001 were indicated as “**”, “***” respectively. (D) Gene signature enrichment analysis of MHC-II antigen presentation genes in primary and escaped tumor DCs. Rest: resting spleen DCs isolated from healthy mice; Acti: spleen DCs isolated from LPS and ovalbumin active mice; Pri: primary tumor DCs isolated from KR158B tumor with no treatment (as indicated in Figure 1A); Esc: escaped tumor DCs isolated from KR158B tumor escaped from ACT treatment (as indicated in Figure 1A). Figure S2: (A) DC subtyping in primary and escaped tumors by flowcytometry. (B) CD45^+^ population ratio in primary and escaped tumor cells by flowcytometry. (C–G) GSEA for pathways enriched in different DC groups as indicated. Only pathways have FDR q value < 0.1 were shown. (H) Gene editing efficiency of HIF1α in BMDCs by Inference of CRISPR Edits (ICE) analysis. (I) HIF1α knock-out validation by Immunofluorescence. Scale bar = 40 µm. Red = nuclear stain (nucspot 750/780). Green = HIF1a (AF488). (J) Gene expression level by RT-qPCR in control and HIF1α knockout BMDCs under normoxia and hypoxia using a different HIF1α targeting sgRNA from Figure 3E. The mean ± SD is indicated. Significance in (A,B) was calculated using a two-sided unpaired *t*-test. Significance in (J) was calculated using one-way anova. *n* = 3, *p* value < 0.05, 0.01, 0.001 were indicated as “*”, “**”, “***” respectively. For Figure S2A,B, data were collected from different mouse tissue. For Figure S2I, data were collected from 3 independent repeats. Figure S3: (A) FoxP3^+^ population ratio in GZMB^+^PD1^−^ CD4 T cells from primary and ACT escaped tumors determined by flowcytometry. (B) FoxP3^+^ population ratio in GZMB^+^PD1^−^ CD4 T cells from primary and ACT escaped tdLNs determined by flowcytometry. The mean ± SD is indicated. Significance in (A,B) was calculated using a two-sided unpaired *t*-test. *n* = 6 for primary group, *n* = 5 for escaped group, two of tdLNs from primary group was not included as poor sample preparation. Data was collected from different mouse tissue. (C) FoxP3^+^ CD4 T cells in primary and escaped tumors were determined by flowcytometry. (D) FoxP3^+^ CD4 T cells in tdLNs from endpoint approaching primary tumor bearing mice and ACT escaped mice were determined by flowcytometry. The mean ± SD is indicated. Significance in (C,D) was calculated using a two-sided unpaired *t*-test. *p* value < 0.001 was indicated as “***”. Data was collected from multiple mice tissue. (E) CD4 T cells are not induced into more FoxP3 positive cells after treatment of DC culture conditioned medium under hypoxia compared with normoxia. BMDCs were treated for 24 h under hypoxia or normoxia. Conditioned mediums were collected for treating CD4 T cells in vitro for 24 h. Flowcytometry was performed to determine FoxP3 signal. Figure S4: (A) Correlation plot for HIF1α and Mki67 gene expression in different regions of KR158B-luc tumor brain from combined and individual 9 Gy + HSC, HSC + αPD1, ACT groups. Each dot represents the gene expression from one selected region in spatial transcriptomics data. Correlation coefficient and p value were calculated by pearson method. (B) Repeat of HIF1α protein levels in BMDCs treated by conditioned medium from different brain slice culture as indicated in Figure 4B were determined by Western blot. Relative expression of HIF1α to ACTIN is indicated in number. Figure S5: (A) Gating strategy for DC FACS sorting. (B) Gating strategy for DC subtyping. (C) Gating strategy for T cell phenotyping in Figure 1. Table S1: T cell phenotyping. Table S2: T cell proliferation. Table S3: T cell activation. Table S4: Primers used in the manuscript.

## Abbreviations

ACT	Adoptive cellular transfer
GBM	Glioblastoma
DC	Dendritic cell
BMDC	Bone marrow-derived dendritic cell
HSC	hematopoietic stem cell
OVA	ovalbumin
LPS	Lipopolysaccharide
tdLN	Tumor-draining lymph node

## References

[1] Ostrom Q.T., Patil N., Cioffi G., Waite K., Kruchko C., Barnholtz-Sloan J.S. (2020). CBTRUS Statistical Report: Primary Brain and Other Central Nervous System Tumors Diagnosed in the United States in 2013–2017. Neuro-Oncology.

[2] Sampson J.H., Gunn M.D., Fecci P.E., Ashley D.M. (2020). Brain immunology and immunotherapy in brain tumours. Nat. Rev. Cancer.

[3] Liu Y., Zhou F., Ali H., Lathia J.D., Chen P. (2024). Immunotherapy for glioblastoma: Current state, challenges, and future perspectives. Cell Mol. Immunol..

[4] Dan J., Laura Falceto F., Catherine F., Ahmed L. (2023). Perspective Chapter: Dendritic Cells in The Tumor Microenvironment. Tumor Microenvironment—New Insights.

[5] Hubbe M.L., Jaehger D.E., Andresen T.L., Andersen M.H. (2020). Leveraging Endogenous Dendritic Cells to Enhance the Therapeutic Efficacy of Adoptive T-Cell Therapy and Checkpoint Blockade. Front. Immunol..

[6] Zong J., Keskinov A.A., Shurin G.V., Shurin M.R. (2016). Tumor-derived factors modulating dendritic cell function. Cancer Immunol. Immunother..

[7] Hegde S., Krisnawan V.E., Herzog B.H., Zuo C., Breden M.A., Knolhoff B.L., Hogg G.D., Tang J.P., Baer J.M., Mpoy C. (2020). Dendritic Cell Paucity Leads to Dysfunctional Immune Surveillance in Pancreatic Cancer. Cancer Cell.

[8] Ma Y., Shurin G.V., Peiyuan Z., Shurin M.R. (2013). Dendritic cells in the cancer microenvironment. J. Cancer.

[9] Ruan K., Song G., Ouyang G. (2009). Role of hypoxia in the hallmarks of human cancer. J. Cell Biochem..

[10] Taylor C.T., Colgan S.P. (2017). Regulation of immunity and inflammation by hypoxia in immunological niches. Nat. Rev. Immunol..

[11] Mancino A., Schioppa T., Larghi P., Pasqualini F., Nebuloni M., Chen I.H., Sozzani S., Austyn J.M., Mantovani A., Sica A. (2008). Divergent effects of hypoxia on dendritic cell functions. Blood.

[12] Tran C.W., Gold M.J., Garcia-Batres C., Tai K., Elford A.R., Himmel M.E., Elia A.J., Ohashi P.S. (2020). Hypoxia-inducible factor 1 alpha limits dendritic cell stimulation of CD8 T cell immunity. PLoS ONE.

[13] Sanmarco L.M., Rone J.M., Polonio C.M., Fernandez Lahore G., Giovannoni F., Ferrara K., Gutierrez-Vazquez C., Li N., Sokolovska A., Plasencia A. (2023). Lactate limits CNS autoimmunity by stabilizing HIF-1alpha in dendritic cells. Nature.

[14] Liu J., Zhang X., Chen K., Cheng Y., Liu S., Xia M., Chen Y., Zhu H., Li Z., Cao X. (2019). CCR7 Chemokine Receptor-Inducible lnc-Dpf3 Restrains Dendritic Cell Migration by Inhibiting HIF-1alpha-Mediated Glycolysis. Immunity.

[15] Kohler T., Reizis B., Johnson R.S., Weighardt H., Forster I. (2012). Influence of hypoxia-inducible factor 1alpha on dendritic cell differentiation and migration. Eur. J. Immunol..

[16] Wildes T.J., Dyson K.A., Francis C., Wummer B., Yang C., Yegorov O., Shin D., Grippin A., Dean B.D., Abraham R. (2020). Immune Escape After Adoptive T-cell Therapy for Malignant Gliomas. Clin. Cancer Res..

[17] Reilly K.M., Loisel D.A., Bronson R.T., McLaughlin M.E., Jacks T. (2000). Nf1;Trp53 mutant mice develop glioblastoma with evidence of strain-specific effects. Nat. Genet..

[18] Figg J., Chen D., Falceto Font L., Flores C., Jin D. (2024). In vivo mouse models for adult brain tumors: Exploring tumorigenesis and advancing immunotherapy development. Neuro Oncol..

[19] Flores C.T., Wildes T.J., Drake J.A., Moore G.L., Dean B.D., Abraham R.S., Mitchell D.A. (2018). Lin(−)CCR2(+) hematopoietic stem and progenitor cells overcome resistance to PD-1 blockade. Nat. Commun..

[20] Warwick C.A., Usachev Y.M. (2017). Culture, Transfection, and Immunocytochemical Analysis of Primary Macrophages. Methods Mol. Biol..

[21] Flores C., Pham C., Snyder D., Yang S., Sanchez-Perez L., Sayour E., Cui X., Kemeny H., Friedman H., Bigner D.D. (2015). Novel role of hematopoietic stem cells in immunologic rejection of malignant gliomas. Oncoimmunology.

[22] Wildes T.J., Grippin A., Dyson K.A., Wummer B.M., Damiani D.J., Abraham R.S., Flores C.T., Mitchell D.A. (2018). Cross-talk between T Cells and Hematopoietic Stem Cells during Adoptive Cellular Therapy for Malignant Glioma. Clin. Cancer Res..

[23] Chow A., Perica K., Klebanoff C.A., Wolchok J.D. (2022). Clinical implications of T cell exhaustion for cancer immunotherapy. Nat. Rev. Clin. Oncol..

[24] Rosenberg S.A. (2011). Cell transfer immunotherapy for metastatic solid cancer--what clinicians need to know. Nat. Rev. Clin. Oncol..

[25] Wrzesinski C., Paulos C.M., Gattinoni L., Palmer D.C., Kaiser A., Yu Z., Rosenberg S.A., Restifo N.P. (2007). Hematopoietic stem cells promote the expansion and function of adoptively transferred antitumor CD8 T cells. J. Clin. Investig..

[26] Dudley M.E., Yang J.C., Sherry R., Hughes M.S., Royal R., Kammula U., Robbins P.F., Huang J., Citrin D.E., Leitman S.F. (2008). Adoptive cell therapy for patients with metastatic melanoma: Evaluation of intensive myeloablative chemoradiation preparative regimens. J. Clin. Oncol..

[27] Gattinoni L., Powell D.J., Rosenberg S.A., Restifo N.P. (2006). Adoptive immunotherapy for cancer: Building on success. Nat. Rev. Immunol..

[28] Xiao Z., Wang R., Wang X., Yang H., Dong J., He X., Yang Y., Guo J., Cui J., Zhou Z. (2023). Impaired function of dendritic cells within the tumor microenvironment. Front. Immunol..

[29] DeVito N.C., Plebanek M.P., Theivanthiran B., Hanks B.A. (2019). Role of Tumor-Mediated Dendritic Cell Tolerization in Immune Evasion. Front. Immunol..

[30] Cowburn A.S., Crosby A., Macias D., Branco C., Colaco R.D., Southwood M., Toshner M., Crotty Alexander L.E., Morrell N.W., Chilvers E.R. (2016). HIF2alpha-arginase axis is essential for the development of pulmonary hypertension. Proc. Natl. Acad. Sci. USA.

[31] Anand R.J., Gribar S.C., Li J., Kohler J.W., Branca M.F., Dubowski T., Sodhi C.P., Hackam D.J. (2007). Hypoxia causes an increase in phagocytosis by macrophages in a HIF-1alpha-dependent manner. J. Leukoc. Biol..

[32] Kushwah R., Hu J. (2011). Role of dendritic cells in the induction of regulatory T cells. Cell Biosci..

[33] Tibbs E., Kandy R.R.K., Jiao D., Wu L., Cao X. (2023). Murine regulatory T cells utilize granzyme B to promote tumor metastasis. Cancer Immunol. Immunother..

[34] Cao X., Cai S.F., Fehniger T.A., Song J., Collins L.I., Piwnica-Worms D.R., Ley T.J. (2007). Granzyme B and perforin are important for regulatory T cell-mediated suppression of tumor clearance. Immunity.

[35] Steindl A., Valiente M. (2025). Potential of ex vivo organotypic slice cultures in neuro-oncology. Neuro Oncol..

[36] Maldonado R.A., von Andrian U.H. (2010). How tolerogenic dendritic cells induce regulatory T cells. Adv. Immunol..

[37] Li M.O., Flavell R.A. (2008). Contextual regulation of inflammation: A duet by transforming growth factor-beta and interleukin-10. Immunity.

