# Supplementary material for: Dendritic Cell Dysfunction Underlies Immune Escape After Adoptive Cellular Therapy in Glioblastoma
Source: Cancers (Basel). 2026 Aug 18;18(16):2669. doi: 10.3390/cancers18162669 (PMC13510367; doi:10.3390/cancers18162669)
Supplement: Supplementary file 1 [file cancers-18-02669-s001.zip › Supplementary Data.pdf]

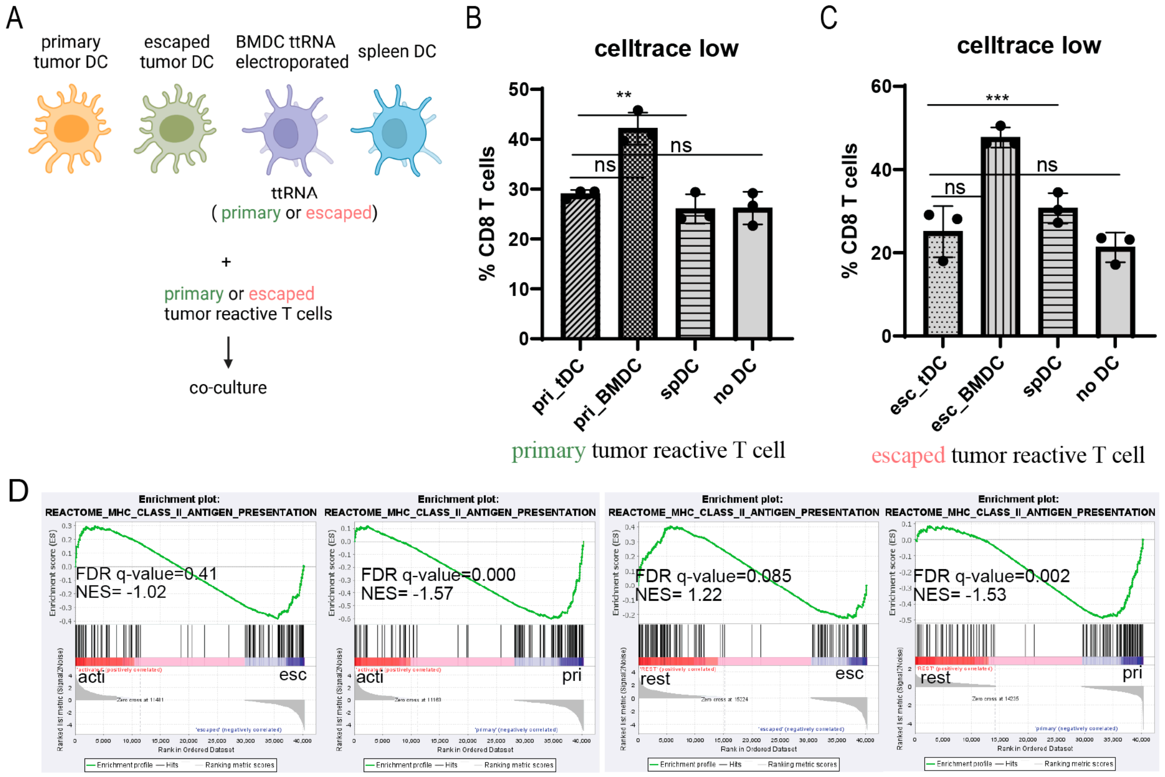

Figure S1

A. Illustration of experimental design for T cell activation assay (Figure S1B).

B. Fast proliferating CD8 T cells with low celltrace signal in co-culture with primary tumor reactive T cells were determined by flowcytometry. pri-tDC: primary tumor DC; pri_BMDC: BMDC electroporated with primary tumor RNA; spDC: spleen DC from naïve mice; no DC: no DCs were added

C. Fast proliferating CD8 T cells with low celltrace signal in co-culture with escaped tumor reactive T cells were determined by flowcytometry. esc-tDC: escaped tumor DC; esc_BMDC: BMDC electroporated with escaped tumor RNA; spDC: spleen DC from naïve mice ;no DC: no DCs were added

The mean ± SD is indicated. One way ANOVA (more than two groups) were performed by Graphpad prism 10.0 for (B,C). *n* = 3,p value < 0.01,0.001 were indicated as "**", "***" respectively.

D. Gene signature enrichment analysis of MHC-II antigen presentation genes in primary and escaped tumor DCs. Rest: resting spleen DCs isolated from healthy mice; Acti: spleen DCs isolated from LPS and ovalbumin active mice; Pri: primary tumor DCs isolated from KR158B tumor with no treatment (as indicated in Figure 1A); Esc: escaped tumor DCs isolated from KR158B tumor escaped from ACT treatment (as indicated in Figure 1A).

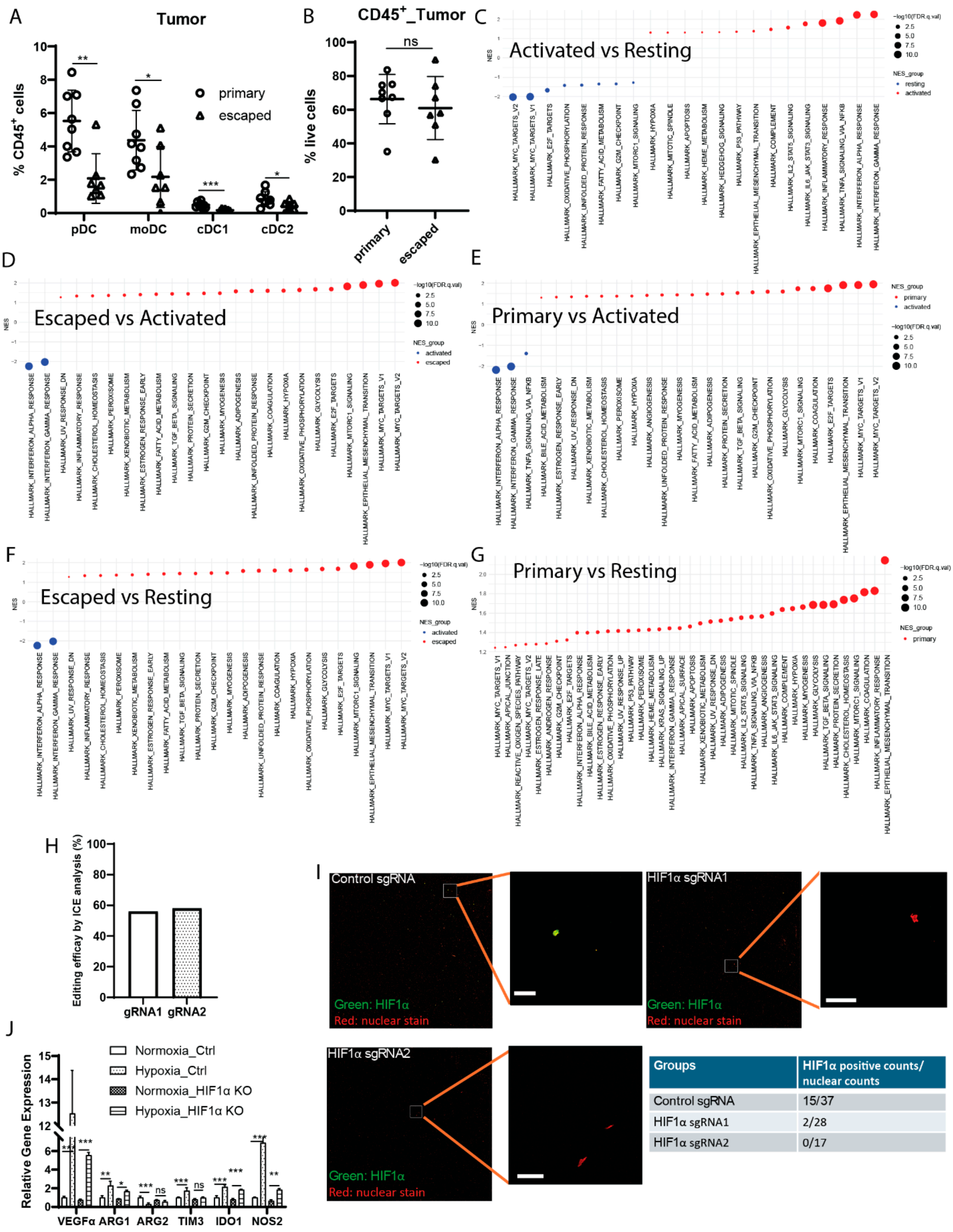

A
Tumor
% CD45+ cells
primary
escaped
pDC
moDC
cDC1
cDC2
B
CD45+_Tumor
% live cells
ns
primary
escaped
C
Activated vs Resting
D
Escaped vs Activated
E
Primary vs Activated
F
Escaped vs Resting
G
Primary vs Resting
H
Editing efficacy by ICE analysis (%)
gRNA1
gRNA2
I
Control sgRNA
HIF1α sgRNA1
HIF1α sgRNA2
Green: HIF1α
Red: nuclear stain
Groups | HIF1α positive counts/ nuclear counts
Control sgRNA | 15/37
HIF1α sgRNA1 | 2/28
HIF1α sgRNA2 | 0/17
J
Relative Gene Expression
Normoxia_Ctrl
Hypoxia_Ctrl
Normoxia_HIF1α KO
Hypoxia_HIF1α KO
VEGFα
ARG1
ARG2
TIM3
IDO1
NOS2

Figure S2

A. DC subtyping in primary and escaped tumors by flowcytometry
B. CD45+ population ratio in primary and escaped tumor cells by flowcytometry

C–G. GSEA for pathways enriched in different DC groups as indicated. Only pathways have FDR q value <0.1 were shown.

H. Gene editing efficiency of HIF1α in BMDCs by Inference of CRISPR Edits (ICE) analysis.
I. HIF1α knock-out validation by Immunofluorescence. Scale bar= 40 μm. Red = nuclear stain (nucspot 750/780). Green = HIF1a (AF488)
J. Gene expression level by RT-qPCR in control and HIF1α knockout BMDCs under normoxia and hypoxia using a different HIF1α targeting sgRNA from Figure 3E.

The mean ± SD is indicated. Significance in (A,B) was calculated using a two-sided unpaired *t*-test. Significance in (I) was calculated using one-way anova. $n = 3$, p value < 0.05, 0.01, 0.001 were indicated as “*”, “**”, “***” respectively. For Figure S2A,B, data were collected from different mouse tissue. For Figure S2I, data were collected from 3 independent repeats.

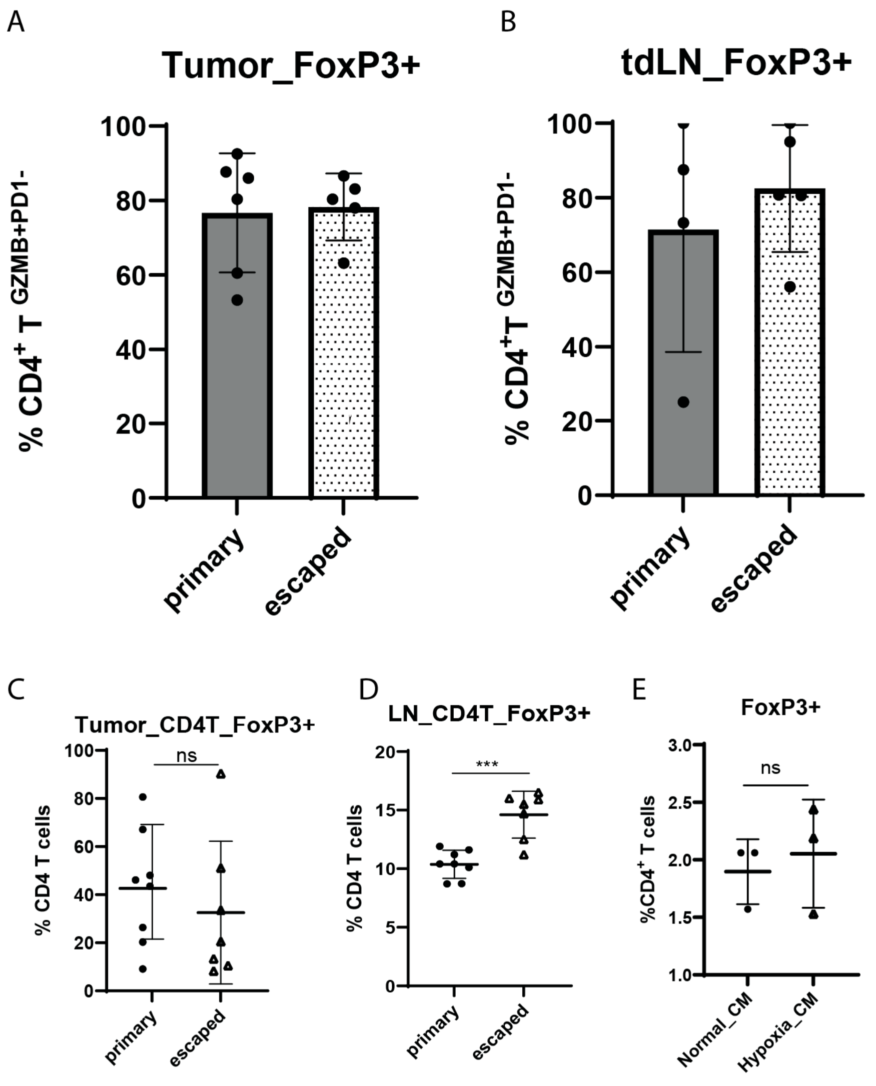

Figure S3

A. FoxP3+ population ratio in GZMB⁺PD1⁻ CD4 T cells from primary and ACT escaped tumors determined by flowcytometry.

B. FoxP3⁺ population ratio in GZMB⁺PD1⁻ CD4 T cells from primary and ACT escaped tdLNs determined by flowcytometry.

The mean ± SD is indicated. Significance in (A,B) was calculated using a two-sided unpaired *t*-test. *n* = 6 for primary group, *n* = 5 for escaped group, two of tdLNs from

primary group was not included as poor sample preparation. Data was collected from different mouse tissue.

C. $FoxP3^+$ CD4 T cells in primary and escaped tumors were determined by flowcytometry.

D. $FoxP3^+$ CD4 T cells in tdLNs from endpoint approaching primary tumor bearing mice and ACT escaped mice were determined by flowcytometry.

The mean ± SD is indicated. Significance in (C,D) was calculated using a two-sided unpaired *t*-test. p value < 0.001 were indicated as “***”. Data was collected from multiple mice tissue.

E. CD4 T cells are not induced into more FoxP3 positive cells after treatment of DC culture conditioned medium under hypoxia compared with normoxia. BMDCs were treated for 24 h under hypoxia or normoxia. Conditioned mediums were collected for treating CD4 T cells in vitro for 24 h. Flowcytometry was performed to determine FoxP3 signal.

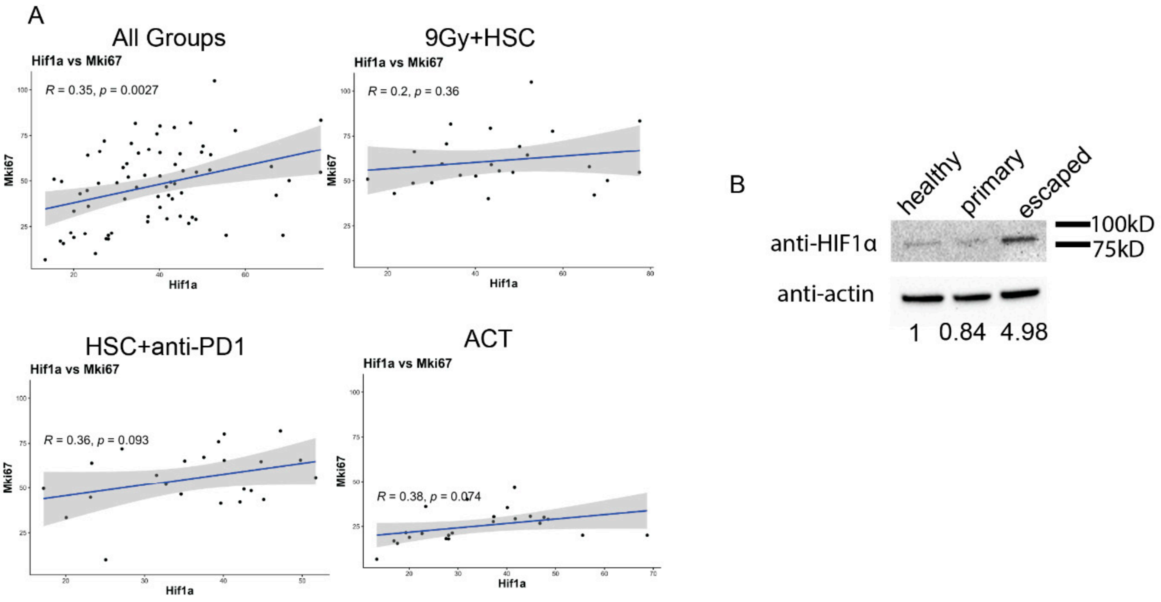

Figure S4

A. Correlation plot for HIF1α and Mki67 gene expression in different regions of KR158B-luc tumor brain from combined and individual 9 Gy + HSC, HSC + αPD1, ACT groups. Each dot represents the gene expression from one selected region in spatial transcriptomics data. Correlation coefficient and p value were calculated by pearson method.

B. Repeat of HIF1α protein levels in BMDCs treated by conditioned medium from different brain slice culture as indicated in Figure 4B were determined by Western blot. Relative expression of HIF1α to ACTIN is indicated in number.

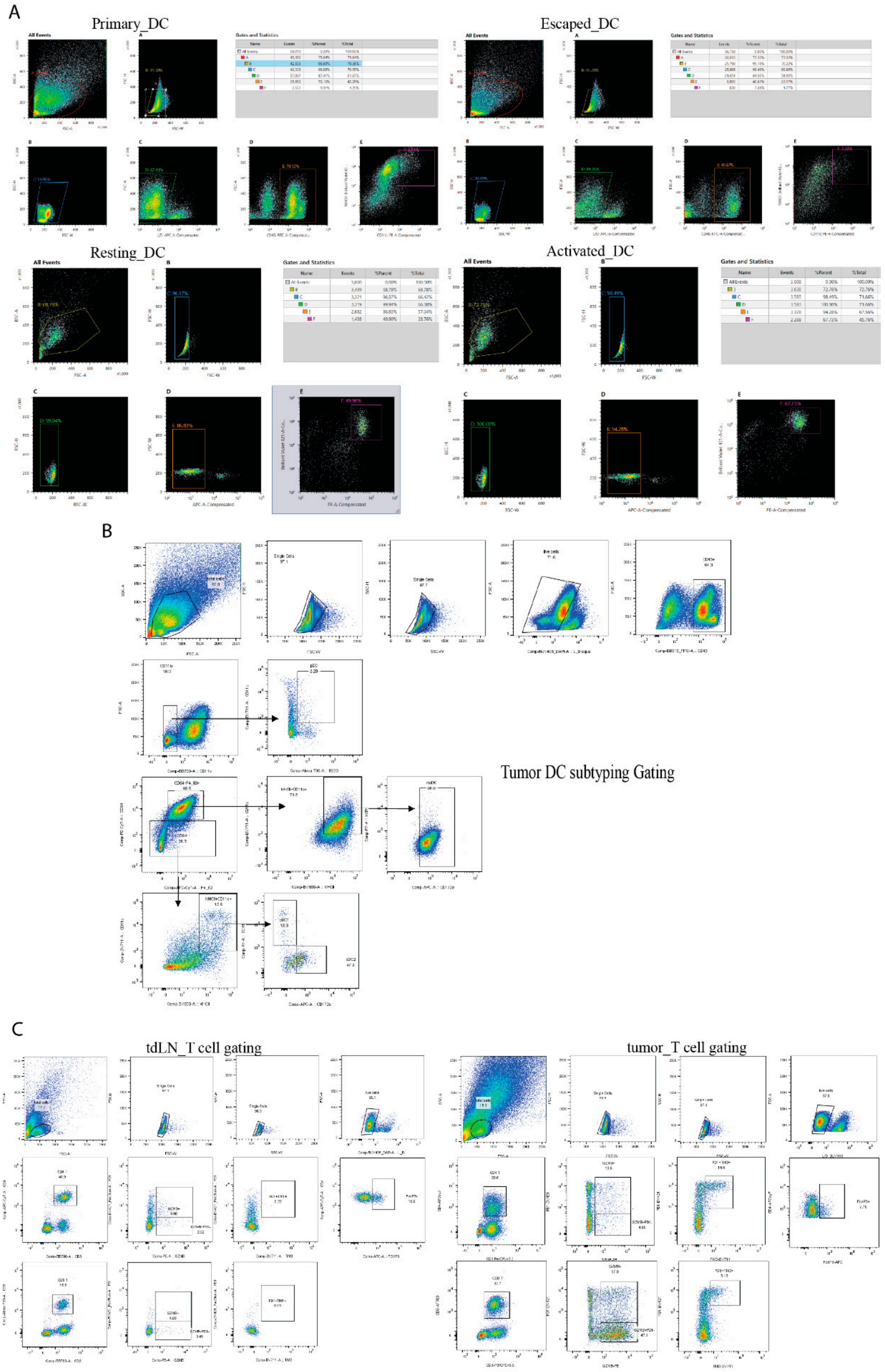
A
Primary_DC
Escaped_DC
Resting_DC
Activated_DC
B
Tumor DC subtyping Gating
C
tdLN_T cell gating
tumor_T cell gating

Figure S5

A. Gating strategy for DC FACS sorting
B. Gating strategy for DC subtyping
C. Gating strategy for T cell phenotyping in Figure 1
