# Supplementary material for: Dendritic Cell Dysfunction Underlies Immune Escape After Adoptive Cellular Therapy in Glioblastoma
Source: Cancers (Basel). 2026 Aug 18;18(16):2669. doi: 10.3390/cancers18162669 (PMC13510367; doi:10.3390/cancers18162669)
Supplement: Supplementary file 1 [file cancers-18-02669-s001.zip › Supplementary Tables.pdf]

Table S1. T cell phenotyping.

Antibody	Dilution	Cat#
CD45.1-PEcy7	1:100	Biolegend, 110730
CD8-AF700	1:200	Biolegend, 100729
CD4-APCcy7	1:200	Biolegend, 100413
CD3-PerCPcy5.5	1:100	Biolegend, 100218
GZMB-PE	1:200	Biolegend, 372207
PD1-BV421	1:200	Biolegend, 135221
TIM3-BV711	1:200	Biolegend, 119727
FoxP3-AF647	1:200	Biolegend, 126408
L/D-aqua	1:300	ThermoFisher, L34957
GFP		

Table S2. T cell proliferation.

Antibody	Dilution	Cat#
CD8-FITC	1:200	Biolegend,
CD4-APCcy7	1:200	Biolegend, 100413
CD3-PerCPcy5.5	1:100	Biolegend, 100218
Celltrace-Violet	1:1000	ThermoFisher, C34557
L/D-aqua	1:300	ThermoFisher, L34957

Table S3. T cell activation.

Antibody	Dilution	Cat#
CD8-PEcy7	1:200	BD, 552877
CD4-APCcy7	1:200	Biolegend, 100413
CD3-PerCPcy5.5	1:100	Biolegend, 100218
CD62L-FITC	1:200	Biolegend, 104406
CD44-APC	1:200	Biolegend,103012
CD69-PE	1:200	Biolegend,104507
L/D-near IR	1:1000	ThermoFisher, L34975

Table S4. Primers used in the manuscript.

Name	Oligo seq	experiment
NOS2-qF	ACGAGACGGATAGGCAGAGA	RT-qPCR
NOS2-qR	GCACATGCAAGGAAGGGAAC	
Arg1-qF	GTACATTGGCTTGCGAGACG	
Arg1-qR	GCCAATCCCCAGCTTGTCTA	
IDO1-qF	CCAGTGCAGTAGAGCGTCAA	
IDO1-qR	TCTGGGTCCACAAAGTCACG	
Havcr2/TIM3-qF	GGGCAAGGGATTCTGTCCTT	
Havcr2/TIM3-qR	TCCTTTGTTGAGATCGCCCT	
mActb-qF	GATATCGCTGCGCTGGTC	
mActb-qR	CATTCCCACCATCACACCCT	
TGFB1-qF	CCT GAG TGG CTG TCT TTT GA	
TGFB1-qR	CGT GGA GTT TGT TAT CTT TGC TG	
VEGFa-qF	GGCAGCTTGAGTTAAACGAAC	
VEGFa-qR	TGGTGACATGGTTAATCGGTC	
ARG2-qF	GATCTCTGTGTCATCTGGGTTG	
ARG2-qR	AATCCTGGCAGTTGTGGTAC	
LDHA_qF	GTCTCCCTGAAGTCTCTTAACC	
LDHA_qR	ACCTTTCAGCTTGATCACCTC	
HIF1a-qF	ACCTGGCAATGTCTCCTTTAC	
HIF1a-qR	CCAGTGACTCTGGACTTGATTC	
HIF1a-KO1-F2	GAGATGCAGCAAGATCTCGG	ICE
HIF1a-KO1-R2	GATACCCTCTTCTGGCCTCC	
HIF1a-KO2-F2	TGCAGGTGGTCTAGACAGTG	
HIF1a-KO2-R2	TCA CCA TTT CTG TGT GTA AGC A	
HIF1alpha_sgRNA1	CAAGATGTGAGCTCACATTG	Knock out
HIF1alpha_sgRNA2	GCTAACAGATGACGGCGACA	
